# Supplementary material for: Kangaroo Mother Care implementation research to develop models for accelerating scale-up in India and Ethiopia: study protocol for an adequacy evaluation
Source: BMJ Open. 2019 Nov 21;9(11):e025879. doi: 10.1136/bmjopen-2018-025879 (PMC6886988; doi:10.1136/bmjopen-2018-025879)
Supplement: Author's manuscript [file bmjopen-2018-025879.draft_revisions.pdf]

BMJ Open

BMJ Open is committed to open peer review. As part of this commitment we make the peer review history of every article we publish publicly available.

When an article is published we post the peer reviewers' comments and the authors' responses online. We also post the versions of the paper that were used during peer review. These are the versions that the peer review comments apply to.

The versions of the paper that follow are the versions that were submitted during the peer review process. They are not the versions of record or the final published versions. They should not be cited or distributed as the published version of this manuscript.

BMJ Open is an open access journal and the full, final, typeset and author-corrected version of record of the manuscript is available on our site with no access controls, subscription charges or pay-per-view fees (http://bmjopen.bmj.com).

If you have any questions on BMJ Open's open peer review process please email info.bmjopen@bmj.com

BMJ Open

Kangaroo Mother Care Implementation Research to Develop Models for Accelerating Scale-up in India and Ethiopia: Study Protocol for an Adequacy Evaluation

Journal:	*BMJ Open*
Manuscript ID	bmjopen-2018-025879
Article Type:	Protocol
Date Submitted by the Author:	07-Nov-2018

Complete List of Authors:	Bahl, Rajiv; World Health Organization<br>McRae, Daphne; University of British Columbia, School of Population and Public Health; University of Saskatchewan College of Medicine, Saskatchewan Population Health and Evaluation Research Unit<br>Cranmer, John; Emory University, Nell Hodgson Woodruff School of Nursing<br>Gobezayehu, Abebe; Emory University Ethiopia<br>Chan, Grace; Harvard Medical School; Boston Children's Hospital , Division of Medicine Critical Care<br>Mariam, Damen; Addis Ababa University School of Public Health<br>Tadele, Henok; Hawassa University<br>Mazumder, Sarmila; Society for Applied Studies, Centre for Health Research and Development<br>Mony, Prem; St. John's Research Institute, St. John's National Academy of Health Sciences, Division of Epidemiology & Population Health<br>Kumar, Vishwajeet ; St. John's Medical College & Research Institute, Community Health and Epidemiology<br>Martines, Jose; Universitetet i Bergen Senter for internasjonal helse, Centre for Intervention Science in Maternal and Child Health<br>Pn Rao, Suman; St John’s Medical College Hospital, Department of Neonatology<br>Darmstadt, G; Stanford University School of Medicine, Department of Pediatrics<br>Krishna, Raghav ; Community Empowerment Lab, Global Health<br>Kajal, FNU; Indian Administrative Service, National Health Mission<br>Pileggi-Castro, Cynthia; Centro de Referência da Saúde da Mulher de Ribeirão Preto, Maternidade<br>Sibley, Lynn M.; Emory University School of Public Health, Global Health<br>Jayanna, K; Karnataka Health Promotion Trust, Bangalore, Karnataka, India, Quality Improvement<br>Mohan, H L; Karnataka Health Promotion Trust, Community Mobilization<br>Kar, Arin; Karnataka Health Promotion Trust, Department of Health & Family Welfare<br>Amin, Hajira; Emory University , M&E<br>Upadhyay, Ravi; Centre for Health Research and Development,<br>Madhur, Tarun; Society for Applied Studies, Centre for Health Research and Development<br>Jadaun, Arun; Society for Applied Studies, Centre for Health Research and Development

Bhandari, Nita; Society for applied studies, Centre for Health Research and Development
Kumar, Aarti; Foundation for Community Empowerment, Global Health
Estifanos, Abiy; Addis Ababa University School of Public Health
Fikre, Addisalem; Addis Ababa University School of Public Health
Alemu, Hilbret; John Snow Inc, Urban Health
Tariku, Abraham; Federal Ministry of Health
Duguma, Dereje; Oromia Regional Health Bureau
Medhanyie , Araya; Mekelle University College of Health Sciences, School of Public Health
Abay, Tedros; Mekelle University College of Health Sciences, Department of Pediatrics and Child Health
Beyene , Selemawit; Mekelle University College of Health Sciences, School of Public Health
Gebregizabher , Fisseha; Tigray Regional Health Bureau
Hadush, Marta; Mekelle University College of Health Sciences, Department of Pediatrics and Child Health
Lemango , Ephrem; Federal Ministry of Health , Maternal and Child Health Directorate
Tadesse, Birkneh; Hawassa University College of Medicine and Health Sciences, Department of Pediatrics and Child Health
Asefa, Anteneh; Hawassa University College of Medicine and Health Sciences, School of Public Health
Beyene, Habtamu; SNNPR Regional Health Bureau
Worku, Bogale; Pediatrics Society; Addis Ababa University
Muleta, Mekonnen; Private Consultant
Brune, Thomas; Karloniska Institute, Department of Neonatology
Singhal, Nalini; University of Calgary Cumming School of Medicine, Department of Neonatology
Aziz, Khalid; University of Alberta, Department of Neonatology
Belew, Mulusew; Emory University, Amhara Regional Office
Andualem, Bizuayehu; Amhara National Regional Health Bureau
M, Rajini; Government of Karnataka, Department of Health & Family Welfare

Keywords: kangaroo-mother care method, low birth weight, newborn

SCHOLARONE™ Manuscripts

TITLE PAGE

KANGAROO MOTHER CARE IMPLEMENTATION RESEARCH TO DEVELOP MODELS FOR ACCELERATING SCALE-UP IN INDIA AND ETHIOPIA: STUDY PROTOCOL FOR AN ADEQUACY EVALUATION

John Cranmer, Abebe Gebremariam Gobezayehu, Grace J Chan, Damen Haile Mariam, Henok Tadele, Araya Abrha Medhanyie, Sarmila Mazumder, Prem K. Mony, Vishwajeet Kumar, Rajiv Bahl on behalf of the KMC Scale-Up Study Group*

Corresponding author:

Rajiv Bahl – Coordinator MRD, Department of Maternal, Newborn, Child and Adolescent Health, World Health Organization. Geneva 1211, Switzerland (email: bahlr@who.int)

Phone number: +4179 4755492

Financial disclosure statement: The authors have no financial relationship relevant to this article to disclose

Funding / Support: The World Health Organization, the sponsor, will fund the study with financial support from the Bill and Melinda Gates Foundation. WHO facilitated the preparation of the protocol, will monitor implementation and analysis of data. It will also facilitate the writing of the report and participate in the decision to submit the report for publication. The study teams will have ultimate authority over any of these activities

Potential conflicts of interest: The authors have no conflicts of interest relevant to this article to disclose

Clinical Trial registry name and registration number:

Community Empowerment Laboratory, Uttar Pradesh, India - ISRCTN12286667

St John's National Academy of Health Sciences, Bangalore, India and Karnataka Health Promotion Trust, Bangalore, India - CTRI/2017/07/008988.

Society for Applied Studies, Delhi - NCT03098069

Oromia, Ethiopia - NCT03419416.

Amhara, SNNPR and Tigray, Ethiopia - NCT03506698.

List of abbreviations

CHW- community health worker
HEW- Health extension worker
KMC- Kangaroo mother care
LBW- Low birth weight

For peer review only - http://bmjopen.bmj.com/site/about/guidelines.xhtml

L10K- Last ten kilometers project
NICU- Neonatal intensive care unit
QA- Quality assurance
QI- Quality improvement
SNCU- Special neonatal care unit
SNNPR- Southern Nations Nationalities and People's Region
UP- Uttar Pradesh
WHO- World Health Organization

Short running title: Kangaroo Mother Care implementation research protocol

For peer review only

For peer review only - http://bmjopen.bmj.com/site/about/guidelines.xhtml

Contributor's statement page

All authors contributed the preparation of the manuscript. The specific contributions are listed below:

Conceptualization of the study and first drafting of the manuscript: John Cranmer, Abebe Gebremariam Gobezayehu, Mulusew Lijalem, Grace J Chan, Abiy Seifu Estifano, Damen Haile Mariam, Henok Tadele, Birkneh Tilahun Tadesse, Khalid Aziz, Araya Abrha Medhanyie, Tedros Hailu Abaym, Sarmila Mazumder, Ravi Prakash Upadhyay, Nita Bhandari, Prem K. Mony, Suman Rao, Krishnamurthy Jayanna, Arin Kar, Aarti Kumar, Gary Lee Darmstadt, Vishwajeet Kumar, Rajiv Bahl, Cynthia Pileggi-Castro, Jose Carlos Martines

Subsequent revisions of manuscript drafts, completion of information on study settings and methods: John Cranmer, Abebe Gebremariam Gobezayehu, Mulusew Lijalem Belew, Bizuayehu Gashaw Andualem, Hajira Mohammed Amin, Lyn Sibley, Grace J Chan, Abiy Seifu Estifanos, Addisalem Fikre, Hibret Alemu, Abraham Tariku, Dereje Duguma, Damen Haile Mariam, Henok Tadele, Birkneh Tilahun Tadesse, Anteneh Asefa, Habtamu Beyene, Bogale Worku, Mekonnen Muleta, Thomas Brune, Nalini Singhal and Khalid Aziz, Araya Abrha Medhanyie, Tedros Hailu Abaym, Selemawit Asfaw Beyene, Fisseha Ashebir Gebregizabher, Marta Yemane Hadush, Ephrem Tekle Lemango, Sarmila Mazumder, Ravi Prakash Upadhyay, Tarun Kumar Madhur, Arun Jadaun, Nita Bhandari, Prem K. Mony, Suman Rao, Krishnamurthy Jayanna, Arin Kar, Mohan H. L., Rajini M., Aarti Kumar, Raghav Krishna, Kajal, Gary Lee Darmstadt, Vishwajeet Kumar, Rajiv Bahl, Cynthia Pileggi-Castro, Jose Carlos Martines.

All authors read and approved the final manuscript.

Patients and public were not involved in the development of the protocol.

For peer review only

For peer review only - http://bmjopen.bmj.com/site/about/guidelines.xhtml

ABSTRACT

Introduction: Kangaroo Mother Care (KMC) is the practice of early, continuous and prolonged skin-to-skin contact between the mother and the baby with exclusive breastfeeding. Despite clear evidence of impact in improving survival and health outcomes among low birth weight infants, KMC coverage has remained low and implementation has been limited. Consequently, only a small fraction of newborns that could benefit from KMC receive it.

Methods and analysis: This implementation research project aims to develop and evaluate district-level models for scaling up KMC in India and Ethiopia that can achieve high population coverage. The project includes formative research to identify barriers and contextual factors that affect implementation and utilization of KMC and design scalable models to deliver KMC across the facility-community continuum. This will be followed by implementation and evaluation of these models in routine care settings, in an iterative fashion, with the aim of reaching a successful model for wider district, state and national-level scale-up. Implementation actions would happen at three levels: "pre-KMC facility" - to maximize the number of newborns getting to a facility that provides KMC; "KMC facility" - for initiation and maintenance of KMC; and "post-KMC facility" - for continuation of KMC at home. Stable infants with birth weight <2000 grams and born in the catchment population of the study KMC facilities would form the eligible population. The primary outcome will be coverage of KMC in the preceding 24 hours and will be measured at discharge from the KMC facility and seven days after hospital discharge.

Ethics and dissemination:

Ethics approval was obtained in all the project sites, and at WHO. Results of the project will be submitted to a peer-reviewed journal for publication, in addition to national level dissemination.

Strengths and limitations of the study

- This will be the first implementation research project to develop and evaluate models for achieving high population coverage with KMC.
- The project is a partnership between research groups and local governments, implemented at large scale, covering populations in diverse regions in two countries, Ethiopia and India, of high prevalence of low birth weight.
- This adequacy evaluation includes no comparison group. The models' success in increasing KMC coverage will be assessed through the comparison with data at the beginning of the project. They indicated that KMC is a rare practice in these settings, covering under 5% of low birth weights, and that competing intervention promoting KMC are not currently implemented in study sites.

For peer review only - http://bmjopen.bmj.com/site/about/guidelines.xhtml

INTRODUCTION

Globally, each year 15 million neonates are born preterm and consequently at a high risk of mortality.[1,2] South Asia and sub-Saharan Africa account for almost two-thirds of the world's preterm babies.[3] Kangaroo Mother Care (KMC) is a low-cost intervention involving early, continuous and prolonged skin-to-skin contact between the mother and the baby and exclusive breastfeeding.[4] KMC has been demonstrated to promote physiologic stability, provide a thermally supportive environment, reduce the risk of serious infections, and reduce the mortality among hospitalized, stable preterm and low birth weight (LBW) infants.[5] Despite the benefits, coverage of KMC has remained low and implementation has largely been limited to specialized hospitals.[6-8] As a result, only a small fraction of newborns that could benefit from KMC receive it. A set of seven studies has been planned in Ethiopia and India to develop and test models of scaling-up KMC effectively.

OBJECTIVES OF THE IMPLEMENTATION RESEARCH

Primary Objective: To develop a delivery model that will result in a high coverage (80%+) and quality of KMC for the target population.

Sub-objective A: To design and identify the most promising delivery model through formative research combined with an iterative quality improvement (QI) approach.

Sub-objective B: To implement the most promising model in the selected study facilities and evaluate quality and coverage of KMC in the population they serve.

Secondary Objectives:

To assess:

1. Population-level duration of KMC, i.e. how many days of KMC were received by infants in the population with birth weight <2000gm during the neonatal period
2. Percent of infants with birth weight <2000gm receiving any KMC (any duration in last 24 hours) at 28 days of life
3. Percent of infants <2000gm who are exclusively breastfed at discharge, seven days post discharge and 28 days post discharge
4. Neonatal mortality rate (including early neonatal mortality).
5. Cost of KMC scale up (in some study sites)

For peer review only - http://bmjopen.bmj.com/site/about/guidelines.xhtml

For peer review only

METHODS/ DESIGN

We are undertaking an international, multi-site, implementation study using a mixed methods design that integrates formative and evaluative components into iterative improvement cycles. Our intervention promotes KMC for babies born <2000gm across the community-facility continuum.

Population

The population will be newborn babies with birth weight less than 2000gm born in the geographic area targeted by the intervention. It includes all babies born in health facilities and in the community. For those newborns who are severely sick according to pre-defined criteria (not tolerating oral feeds, severe respiratory distress including, respiratory rate less than 20 breaths per minute, grunting, central cyanosis, severe chest indrawing, convulsions, unconsciousness, and severe hypothermia of less than 32 degrees Celsius), initiation of KMC will be delayed until they have stabilized.

Intervention

The intervention implemented will be the provision of KMC, defined as skin-to-skin contact with the mother or a replacing caregiver, for as long as possible during day and night, with a minimum of at least eight hours over a 24-hour period, along with exclusive breastfeeding or breast milk feeding via tube, spoon or paladai. Based on the available evidence of intervention effectiveness, KMC will only be initiated in health facilities.[5]

Comparison

The performance of the interventions will be assessed against a predefined success criterion of 80% or higher coverage of effective KMC at population level.

Outcomes

The assumption that current coverage with KMC initiated at a health facility is low or negligible in the study population will be confirmed by a baseline assessment of health facilities.

The primary outcome will be effective coverage of KMC, defined as at least eight hours of skin-to-skin contact in combination with exclusive breastfeeding in the preceding 24 hours among infants with birth weight <2000gm. It will be measured:

For peer review only - http://bmjopen.bmj.com/site/about/guidelines.xhtml

- at discharge from the KMC facility (KMC facility is defined as one where staffing, infrastructure and mechanisms are present to initiate and maintain KMC)

- at 7 days after discharge, in the home

- at 28 days of age, in the home

Secondary outcomes include:

- Population-level duration of KMC, i.e. mean of how many days KMC was received by infants in the population with birth weight <2000g during the neonatal period.
- Proportion of infants with birth weight <2000gm receiving any KMC (any duration in last 24 hours) at discharge, seven days post discharge and at 28 days of age
- Proportion of infants with birth weight <2000gm exclusively breastfed (based on 24-hour recall) at discharge, seven days post discharge and at 28 days of age
- Neonatal mortality rate (including early neonatal mortality rate) in infants <2000gm
- Cost of the KMC model (measured in some of the project sites)

Sample Size

We assume that 3-5% of neonates born in Ethiopia and India will be <2000gm and that it will not be possible to evaluate 20% of these newborns because of early death or loss to follow up. A sample size of 310 newborns per site will allow us to estimate 80% coverage of KMC with site-specific absolute precision of ±5%. All the study sites will have sufficient number of births of eligible infants to enroll at least 310 during the evaluation phase. Based on these assumptions, we anticipate the follow-up of a total of at least 2170 newborns <2000gs from the seven sites during the evaluation period.

Study Sites

The study will be conducted in a total of seven sites in Ethiopia and in India. This choice of sites aims to allow the set of studies to reflect regional diversity of the large countries where the studies will be implemented.

Ethiopia

In Ethiopia, newborn mortality remains high at 29 per 1000 live births.[9,10] The Ministry of Health has spearheaded a major campaign to increase facility births which appears effective.[11-15] Results

For peer review only - http://bmjopen.bmj.com/site/about/guidelines.xhtml

from a recent survey (L10K) in 2015 conducted in 115 districts (woredas) in agrarian regions, indicate facility births among women who gave birth in the past 12 months to be around 53%.[11]

Amhara

The study will be conducted in two woredas of West Gojam Zone, two woredas of South Gondar Zone, and Bahir Dar City Adminstration, Amhara Region. In West Gojam zone, the study site includes one primary hospital. In Bahir Dar City Adminstration , the study sites include a large referral hospital, one primary hospital, 19 health centers, 42 health posts and their respective catchment area populations. In South Gondar Zone, the study sites include one general hospital and one primary hospital, 14 health centers and up to 59 health posts affiliated with these health centers, along with their respective catchment area populations.

Oromia – Addis Ababa

The Oromia – Addis Ababa site will implement the study in Akaki Kality sub-city in Addis Ababa and East Shoa zone in Oromia region. Akaki Kality sub-city, one of the 10 sub-cities of Addis Ababa city administration shares border and culture with East Shoa zone of Oromia region. The Akaki Kality sub-city has an estimated population of 180,000 living in 11 woredas in the sub-city.[14] Oromia Regional state has an estimated population of 27 million [14]. The study will be implemented in two woredas: Adami Tulu Woreda in the East Shewa Zone and Arsi Robe Woreda in the Arsi Zone.

SNNPR

SNNPR has a health facility birth rate of 25.2%.[10] The sites proposed for study implementation are Hawassa city administration, Shebedino woreda, and Dale woreda which are situated in Sidama zone. Hawassa is the capital city of SNNPR and its administration consists of eight sub-cities with urban as well as rural kebeles. The two public hospitals in Hawassa city—namely Hawassa University Comprehensive Specialized Hospital and Adare General Hospital—are included in the study. Leku is the capital of Shebedino woreda and consists of urban as well as rural kebeles. Leku Primary Hospital is the study site for Shebedino woreda. Yirgalem is the capital of Dale woreda. Yirgalem General Hospital is the study site for Dale woreda.

Tigray

According to the EDHS 2016, in the Tigray region, 69% of the women who gave birth two years preceding the survey gave birth at health facilities. The study will be implemented in two zones of the region, namely south-east zone and Mekelle special zone. Out of the four districts of the south-

For peer review only - http://bmjopen.bmj.com/site/about/guidelines.xhtml

For peer review only

east zone, two districts namely Enderta and Degu-Tembien woredas will be included in the implementation research. In the selected two districts and Mekelle Zone, there are one comprehensive specialized referral hospital, two general hospitals, one primary hospital, 22 health centers and five private health facilities. All these 31 public and private health facilities provide skilled care at birth (birth assistance) services and will be included in the KMC implementation research.

India

India accounts for one quarter of newborn deaths in the world. Neonatal deaths represent about 50% of under-five deaths and 70% of infant deaths in India.[16] About 27% of all babies are born with low birth weight (<2500g).[17-20]

Haryana

The study will be implemented in one of Haryana's 22 districts named Sonepat. The LBW (<2500 g) rate is 18% in the district.[21,22] Around 5% of all infants weigh less than 2000gm at birth (*information from government records*). The institutional delivery rate is 80.5% and percentage of births conducted by skilled birth attendants is around 90%.[23] Infant mortality rate in the state is 36/1000 live births, which is similar to the Indian national average of 37/1000 live births.[24] The NMR is 28/1000, accounting a major proportion of infant mortality rate.[25]

Karnataka

Infant mortality rate is 28 per 1000 and neonatal mortality rate is 22 per 1000 livebirths.[24,25] The research project will be conducted in the Koppal district, located 400 km away from Bangalore. This is an under-served, high-priority district in the northern region of the state identified as a high priority district by the government.[26] The district has a low birth weight prevalence of about 25% and a neonatal mortality rate of 42 per 1000 live-births. About 80% of births are institutional deliveries.

For peer review only - http://bmjopen.bmj.com/site/about/guidelines.xhtml

Uttar Pradesh (UP)

UP is the most populous state in India and accounts for a quarter of India's burden of neonatal deaths.[25] UP's NMR of 37 per 1000 live births is amongst the highest in the country.[25] The prevalence of low birth weight in UP is 25%.[27] The study will be implemented in the Raebareli district with an institutional delivery rate of 93%. The study will cover the district hospital and ten community health centers, alongwith their catchment blocks.

Ethical Considerations

Since the intervention will be provided by the government as standard of care for LBW infants, there will be no request for consent from individuals to receive the intervention. Individual written informed consent will be requested from mothers, caregivers, and health workers for the collection of information for use in the study. For those who are unable to read, the informed consent form will be read out by the research team member before asking for consent.

Patient and Public Involvement

The question to be addressed by this research project has been identified as a research priority by the governments of Ethiopia and India. They have participated actively in the development of the study plans through the respective ministries of health. Patients and their communities will play a major role in the design of the models to be developed and tested in this study, as described below under the Phase 1 – Formative research. The study results will be disseminated to the communities of the participating districts using the channels available to the health system, as well as to the state and national health authorities and their publication in peer-reviewed journals.

Study approvals

Study approvals were obtained at the local, national and international levels. Ethics approvals have been taken from the ethics committees of the institutions engaged in the research projects and the World Health Organization (WHO) Ethics Committee.

Study implementation strategy

Core Framework for KMC Implementation

KMC promotion will be implemented at three levels (Figure 1):

For peer review only - http://bmjopen.bmj.com/site/about/guidelines.xhtml

"Pre-KMC facility" - the aim at this level is to maximize the proportion of newborns under 2000g to be born in/brought to KMC implementing health facilities.

"KMC facility" – the aim at the facility level is early initiation of KMC and adherence to long duration (>8 hrs) KMC with exclusive breastfeeding within the facility.

"Post-KMC facility" – the aim at the post-facility level is continuation of effective KMC at home through the neonatal period or until the baby no longer accepts it.

Study Teams

The KMC intervention will be delivered by health workers who are part of the health system. The research team's role in implementation will be only supportive. This research team will be comprised of three small teams:

(i) The "program learning team", will be involved with formative research and process-monitoring and will help formulate the intervention and continue to evaluate the process of its implementation.

(ii) The "implementation support team" will guide and support the health system so that the environment is supportive of KMC and health workers implementing the intervention.

(iii) The "outcome evaluation team" will measure the outcomes in the study population acting independently from the other two groups (Table 1).

For peer review only - http://bmjopen.bmj.com/site/about/guidelines.xhtml

Table 1. Description of Study Implementation Teams

	Research			Government Implementation
	Program learning team	Implementation support team	Evaluation team	Health system team
Phase 1	Conduct formative research to develop the initial KMC delivery model.<br><br>Conduct program learning to understand how well the KMC delivery model is working and to redesign the model based on lessons learned.	Support government implementation of KMC.	In the catchment area of the facilities involved in Phase 1, collect quantitative data on:<br>• Births in facilities and community<br>• Births <2000g in facilities and community<br>• Number of newborns receiving KMC at time of discharge from health facility, 7 days post discharge and at 28 days of age<br>• Duration of skin-to-skin care	Deliver and monitor the KMC intervention.
Phase 2	Monitor process indicators to learn how implementation of KMC delivery models happens in all study facilities and identify ways to refine the model based on context-specific findings.	Assume an advisory role to support government implementation of KMC.	In the entire study population, collect quantitative data as above.	Deliver and monitor the KMC intervention.

For peer review only - http://bmjopen.bmj.com/site/about/guidelines.xhtml

Phases of implementation

The implementation will be carried out in two phases (Figure 2).

Phase 1: To Develop a Delivery Model for KMC

KMC Delivery Model Cycles of Improvement

The initial delivery model will be developed based on findings of formative research and synthesis of available literature. Information resulting from program learning from the delivery of this model and outcome measurements will immediately feed into a stage of model review and redesign or refinement, aimed at improving the ability of the model to achieve high coverage. We expect that at least three cycles of model development-implementation-evaluation-refinement will be needed until reaching a scalable model with the desired high coverage characteristics. Each cycle is expected to take approximately three to four months. The first cycle may involve only one facility but the second and third will be larger to include up to one-third of all facilities to be part of the study.

At the end of each cycle, findings will be shared with an advisory group consisting of stakeholders and their input will be sought to improve the model. New iterations of the model will be developed and further refined and agreed upon with advisory groups.

Formative research

The first step in the study will be for the program learning research team to undertake a round of formative research to identify and understand barriers and enablers to implement KMC in the specific local context. Domains of the formative research are presented in Table 2. Based on the knowledge gathered, we will develop an initial model of KMC implementation at the study site.

Implementation of KMC test models

The initial model will be implemented in collaboration with facility and community workers with guidance from the implementation support team. Selection of facilities to implement KMC test models will be based on factors such as number of births, type of health workers available, services delivered (e.g. presence of newborn intensive care units (NICUs) or special care nurseries) so that they capture a major proportion of deliveries in the study district. Implementation will occur at three levels: pre-KMC facility, KMC facility, and post-KMC facility (Figure 1).

For peer review only - http://bmjopen.bmj.com/site/about/guidelines.xhtml

Table 2. Domains of Enquiry for Formative Research

Domains of enquiry	Activities
**KMC Intervention** – to inform the design of the technical package of KMC	• Review KMC scientific literature; existing government guidelines on KMC; experience on implementation of KMC<br>• Develop guidelines and protocols for health staff in facility and community health care workers<br>• Develop communication materials for communities, pregnant women and their families
**Users and implementers** – to understand the barriers and enablers to KMC adoption and adherence	**Mothers/Families**<br>• Explore perceptions of mothers and family members around preterm babies, low birth weight, care-seeking, institutional deliveries, private facilities, perception of quality; understand and explore ideas to generate demand for KMC<br>**Health Workers**<br>• Assess understanding and experience of KMC; understanding of birth weight, prematurity<br>• Assess knowledge, skill, time and motivation for newborn care
**Health systems** – to improve the delivery of KMC at the facility and community level	**Health facilities**<br>• Understand current KMC practices in health facilities; map behaviours that could impact adoption of KMC within these facilities<br>• Design a KMC facility readiness checklist based on local feasibility and provisions<br>• Assess physical infrastructure and resources available for KMC<br>**Link between community and facility**<br>• Assess current engagement between CHWs and health facilities and explore efficient process of engagement<br>• Assess referral system<br>**Assess data needs, measurement systems and analytics support and its compatibility and adaptability for KMC**<br>• Assess existing management information system; assess registries that exist at each level of the health care system<br>• Design and test electronic data management systems to guide and accelerate adoption and improvement of KMC<br>**Leadership and policy makers**<br>• Engage leadership and decision makers for their support of KMC<br>• Assess need of additional workers and current human resource capacity gap<br>**Other stakeholders**<br>• Map and engage all professional, organizational, health system, administrative and social networks that may impede or support KMC adoption and spread in the study area
**Context** – to develop adaptable delivery models	• Understand perceptions of care of small babies and logistical challenges to care of high risk babies within the setting<br>• Explore motivations for facility versus home deliveries<br>• Map relationships between doctors and nurses, CHWs, and mothers/families

For peer review only - http://bmjopen.bmj.com/site/about/guidelines.xhtml

Pre-KMC facility

To effectively implement KMC, KMC eligible newborns need to be identified and cared for in facilities where KMC is initiated. All births need to be identified and babies weighed. Eligible babies, if born outside KMC facilities, will be referred to these facilities through strengthened government transport and linkage mechanisms. The pre-facility level includes efforts to: increase facility deliveries, expand early assessment of those who are delivered at home, so that eligible newborns are referred to a KMC facility, and create a new social norm around the practice of KMC.

KMC facility

Activities at the facility level will serve to strengthen knowledge and skills of health providers, implement KMC protocols and processes within facilities so that they provide high-quality care. All stable newborns born in facilities would be placed in skin-to-skin contact and initiated breastfeeding within the first hour of life. In-born and referred newborns would be screened for KMC eligibility (< 2000gm and stable). If the newborn meets eligibility criteria, KMC would be initiated following the KMC facility protocol. If the newborn is sick, he/she will be stabilized first, as per local protocols, and then reassessed for KMC eligibility and initiation.

During the newborn's facility stay, nurses will do a KMC assessment following a KMC checklist which includes assessment of skin-to-skin contact (number of hours) and breastfeeding, counselling on KMC, and problem-solving if barriers exist. Nurses will document the status of adoption of KMC. At time of discharge, extensive counselling will be given to the mothers and family members to ensure that KMC is continued at home. Facility staff will notify CHWs about the discharge and discuss how to support continued KMC at home.

Post-KMC facility

After discharge from the KMC facility, community health workers(CHWs) shall visit the baby at home within the first 24 hours following the post-facility KMC protocol. CHWs will continue to visit the newborn following the standard national postnatal care home visit schedule. At each visit, the CHW will perform a KMC assessment using the KMC follow-up checklist including assessment of skin-to-skin duration and breastfeeding, counselling on KMC, and problem solving if barriers exist. CHWs will also identify and refer sick newborns or newborns unable to continue KMC to the facility and facilitate the referral process.

For peer review only - http://bmjopen.bmj.com/site/about/guidelines.xhtml

Health systems strengthening

Linkages between the health facility and the community will be strengthened so that KMC initiated at the health facility is continued at home. This will be achieved through establishment of communication systems between the facility and community health workers

Outcome Measurement

The study evaluation team will carry out an independent evaluation of the effectiveness of the model in achieving 80% or higher coverage with KMC among the target population. Data collection will take place at both health facilities and the community in order to measure outcomes.

Phase 2: Implement and Evaluate the chosen KMC Delivery Model

The most promising KMC delivery model will be chosen from Phase 1 based on program learning findings and coverage levels attained. During phase 2, the chosen model will be implemented in all selected facilities and data will be collected in the study area to measure the coverage of the intervention with effective KMC.

Implementation of the KMC Delivery Model

The scalable model will include KMC guidelines, protocols, communication materials, training materials, and tools needed for its successful implementation covering the three above-mentioned levels (pre-KMC facility, KMC facility, and post-KMC facility). Implementation activities will include guideline dissemination; health care provider and CHW training; identification of low birth weight newborns; demand generation; establishment of referral and supervision systems; monitoring; and continuous feedback loops and adaptation.

The KMC delivery model will be implemented by the health system in all study facilities and their catchment areas. The implementation support team will continue to be active but playing only an advisory role to the government implementation teams. There will be ongoing mentorship and quality improvement feedback cycles within each facility..

Process-monitoring

The program learning team will transition to a role of documenting implementation processes by measuring process indicators. Process indicators for KMC implementation have been described in Table 3.

For peer review only - http://bmjopen.bmj.com/site/about/guidelines.xhtml

Table 3. Process Indicators for KMC Implementation

**Process**	Indicator
Identification of pregnant women	• Proportion of pregnant women identified by CHW in the antenatal period
Exposure to KMC priming interactions in the antenatal period	• Proportion of identified pregnant women who received KMC information in the antenatal period from CHWs (either at home visits or during planned community interactions<br>• Proportion of identified pregnant women who received KMC information during ANC visits at health facilities
Delivery in KMC facility	• Proportion of women who deliver in a KMC facility versus in another facility or at home
Weighed at birth	• Proportion of live born babies who were weighed at birth, overall and by place of birth
Identification of newborns <2,000g	• Proportion of babies identified as <2000g, overall and by place of birth
Referral	• Proportion of newborns <2000g who were born at home or in non-KMC facility who were successfully referred to KMC facility for care.
Initiation of KMC	• Proportion of newborns <2000g born in KMC facility who were initiated on KMC<br>• Proportion of newborns <2000g referred to KMC facility who were initiated on KMC
KMC monitoring	• Proportion of KMC initiated newborns monitored by facility staff according to protocol<br>• Proportion of facility KMC newborns discharged according to criteria; left against medical advice; referred out; died before discharge<br>• Duration of stay in facility
KMC follow-up	• Proportion of newborns discharged from facility receiving KMC who received follow-up per protocol

For peer review only - http://bmjopen.bmj.com/site/about/guidelines.xhtml

Outcome Measurement

The outcome measurement team will be responsible for carrying out the evaluation of the effectiveness of the model in achieving 80% or higher coverage with KMC among the entire study population as described above in the outcome measurement section.

Evaluation

Effective KMC coverage is defined as the number of newborns receiving KMC divided by the total number of newborns eligible for KMC (<2000g and stable) in the facilities and in the study population during the defined evaluation period.

To measure the denominator for effective KMC coverage it is preferred that all facility births be identified by the outcome measurement team during facility visits, review of birth registers, and/or calls to the facilities. In addition to this, the outcome measurement team will liaise with community health workers to obtain the number of home births and their birth weights.

To calculate the numerator for effective KMC coverage, the outcome measurement team will quantify the number of newborns less than 2000g who received KMC in the study population. All identified newborns less than 2000g will be followed by the outcome measurement team to collect data on KMC indicators in the facility or at home at four points i.e. day of initiation of KMC, day of discharge from the KMC facility, seven days post-discharge, and at age 28 days. At each time point, the team will attempt to measure KMC based on hospital records, and/or maternal report, if post-discharge.

Field Logistics

Engaging with the government partners

Research teams will engage with the government partners to introduce the KMC initiative and ensure their sustained involvement. Discussions with national and local government officials will be held to revitalize KMC or initiate buy-in.

Community awareness and demand generation activities

Contacts between pregnant women, families and the health system will be utilized to promote awareness of the importance of facility births, of of weighing the baby at birth, of KMC for babies under 2000 gm and of the availability of KMC units in facilities. Various

For peer review only - http://bmjopen.bmj.com/site/about/guidelines.xhtml

sites will identify the most effective strategies for increasing uptake and continuation of KMC within their population and include them as part of their proposed model.

Training

The site teams will receive training on quantitative and qualitative methods. As part of establishing KMC in the selected health facilities and ensuring that it is being practised at home, teams will work along with experienced local paediatricians, faculty from the site-specific institution and the pool of trainers already available in the state. Refresher training sessions will be frequent. A mechanism for follow up with supportive supervision will be set up.

Data Management

Data collected will be linked to the participant identification number. Each research team will have the responsibility for data management for its site. The cleaned evaluation data set will be sent to the data coordinating centre at WHO at monthly intervals for review and feedback. Data quality assurance methods would include online real-time checks for missing values, inconsistency; random spot checks and data validation coupled with independent quarterly quality audits.

Oversight

The Maternal, Newborn, Child and Adolescent Health (MCA) Department of WHO, Geneva will create a Steering Committee and will be responsible for organizing its meetings. The Steering Committee will be the technical body responsible for the oversight of the study.

DISCUSSION

In spite of its scientifically documented benefits, scaling up of KMC has proven an elusive goal for about 40 years. With the increased awareness of the magnitude of newborn mortality and the clear interest of the governments of Ethiopia and India in making KMC available to all newborns of birth weight below 2000g, it is timely to implement this project. It provides a great opportunity to weave KMC within the existing health system and, in collaboration with the government, to find effective models of implementation that can be applied at scale and sustained. The findings from this implementation research project will provide inputs to health policy makers to formulate a KMC implementation guide for state or nationwide scale

For peer review only - http://bmjopen.bmj.com/site/about/guidelines.xhtml

up, if one doesn't yet exist, or to improve if there already exists one. The findings from this implementation study will have implications for additional actions to strengthen the provision of essential newborn care.

Literature on barriers and facilitators of scaling up KMC on a smaller scale within few health facilities is available.[28-30] This project aims to develop models and test them in three to four cycles with an inbuilt iterative learning component, and strong government-research partnerships that will ultimately lead to identification of effective models of scale up in large country-wide settings. While we focus on KMC coverage in our outcomes, our aim is to develop a model that incorporates KMC as an integral component of newborn care so that the KMC scale up is not projected as a vertical program. The experiences from this implementation research will not only inform us on the challenges to scale up KMC and ways to effectively address it but will have the potential to help the design and implementation strategy of future infant and child care programs in a way that their accelerated uptake by health system and the communities is strengthened. The findings of this implementation research will also be useful in other low- and middle-income countries, besides India and Ethiopia.

CONCLUSION

The study has been designed to identify opportunities and constraints in the implementation of KMC at large scale and to develop delivery mechanisms that can reach the desired 80%+ population coverage. The findings from this study will be highly relevant for programmes in low and middle-income countries aiming to improve newborn survival and health.

For peer review only

For peer review only - http://bmjopen.bmj.com/site/about/guidelines.xhtml

***KMC Scale-Up Study Group**

Group authorship by region/state of implementation

ETHIOPIA

Amhara Region –

Principal Investigator: John Cranmer, Emory University, Nell Hodgson Woodruff School of Nursing Atlanta, GA, USA

Principal Investigator: Abebe Gebremariam Gobezayehu, Emory University Ethiopia, Addis Ababa, ET

Mulusew Lijalem Belew, Emory University, Amhara regional office, Bahirdar, Ethiopia

Bizuayehu Gashaw Andualem, Amhara Regional Health Bureau, Bahirdar, Ethiopia

Hajira Mohammed Amin, Emory University Ethiopia Country Office, Addis Ababa, Ethiopia

Lyn Sibley, Emory University, Atlanta, Georgia, USA

Oromia Region –

Principal Investigator: Grace J Chan, Harvard Medical School Boston, MA, USA; Boston Children's Hospital, Division of Medicine Critical Care, Boston, MA, USA

Principal Investigator: Damen Haile Mariam, Addis Ababa University, Addis Ababa, Oromia, ET

Abiy Seifu Estifanos, School of Public Health, Addis Ababa University, Addis Ababa, Ethiopia

Addisalem Fikre, School of Public Health, Addis Ababa University, Addis Ababa, Ethiopia

Hibret Alemu, JSI Urban Health, Addis Ababa, Ethiopia

Abraham Tariku, Federal Ministry of Health, Addis Ababa, Ethiopia

Dereje Duguma, Oromia Regional Health Bureau, Addis Ababa, Ethiopia

SNNPR (Southern Nations Nationalities and Peoples' Region) –

Principal Investigator: Henok Tadele, Hawassa University, Hawassa, Southern Nations, ET

Birkneh Tilahun Tadesse, Hawassa University, Department of Pediatrics and Child Health, Hawassa, Ethiopia

For peer review only - http://bmjopen.bmj.com/site/about/guidelines.xhtml

Anteneh Asefa, Hawassa University, school of Public Health, Hawassa, Ethiopia

Habtamu Beyene, SNNPR Regional Health Bureau, Hawassa, Ethiopia

Bogale Worku, Ethiopian Pediatrics Society and Addis Ababa University, Addis Ababa, Ethiopia

Mekonnen Muleta, Private Consultant, Addis Ababa, Ethiopia

Thomas Brune, Karloniska Institute, Department of Neonatology, Stockholm, Sweden

Nalini Singhal, University of Calgary, Department of Neonatology, Calgary, Canada

Khalid Aziz, University of Alberta, Department of Neonatology, Edmonton, Canada

Tigray Region –

Principal Investigator: Araya Abrha Medhanyie, Mekelle University, Mekelle, Tigray, ET

Tedros Hailu Abaym, Department of Pediatrics and Child Health, Mekelle University, Mekelle, Ethiopia

Selemawit Asfaw Beyene, School of Public Health, Mekelle University, Mekelle, Ethiopia

Fisseha Ashebir Gebregizabher, Tigray Regional Health Bureau, Mekelle, Ethiopia

Marta Yemane Hadush, Department of Pediatrics and Child Health, Mekelle University, Mekelle, Ethiopia

Ephrem Tekle Lemango, Maternal and Child Health Directorate, Federal Ministry of Health, Addis Ababa, Ethiopia

INDIA

Haryana –

Principal Investigator: Sarmila Mazumder, Centre for Health Research and Development, Society for Applied Studies, New Delhi, Delhi, IN

Ravi Prakash Upadhyay, Centre for Health Research and Development, Society for Applied Studies, New Delhi, India

Tarun Kumar Madhur, Centre for Health Research and Development, Society for Applied Studies, New Delhi, India

Arun Jadaun, Centre for Health Research and Development, Society for Applied Studies, New Delhi, India

Nita Bhandari, Centre for Health Research and Development, Society for Applied Studies, New Delhi, India

For peer review only - http://bmjopen.bmj.com/site/about/guidelines.xhtml

Karnataka –

Principal Investigator: Prem K. Mony, St John's Research Insitute, Division of Epidemiology, Biostatistics & Population Health, Bangalore, IN

Suman Rao, St John's Medical College Hospital, Department of Neonatology, Bangalore, IN

Krishnamurthy Jayanna, Karnataka Health Promotion Trust, Bangalore, Karnataka, India, Quality Improvement, Bangalore, IN

Arin Kar, Karnataka Health Promotion Trust, Bangalore

Mohan H. L., Karnataka Health Promotion Trust, Community Mobilization Bangalore, IN

Rajini, M. Department of Health & Family Welfare, Government of Karnataka, Bangalore

Uttar Pradesh –

Principal *Investigator: Vishwajeet Kumar, St. John's Medical College & Research Institute, Community Health and Epidemiology, Bangalore, IN*

Aarti Kumar, Community Empowerment Lab, Global Health, Lucknow, India

Raghav Krishna, Community Empowerment Lab, Global Health, Lucknow, IN

Kajal (no last name), Indian Administrative Service, National Health Mission, Lucknow, IN

Gary Lee Darmstadt, Stanford University School of Medicine, Department of Pediatrics, Stanford, CA, USA

WHO

Principal Investigator: Rajiv Bahl, World Health Organization
20 Avenue Appia, World Health Organization, Geneva, CH

Cynthia Pileggi-Castro, Centro de Referência da Saúde da Mulher de Ribeirão Preto, Maternidade, Ribeirão Preto (SP), BR

Jose Carlos Martines, Universitetet i Bergen Senter for internasjonal helse, Centre for Intervention Science in Maternal and Child Health, Bergen, NO

Study status

WHO approved protocol: Version 4 - 12 May 2016– Protocol ID: ERC 2716

Study implementation beginning: April 2017

Study end: expected March 2019

For peer review only - http://bmjopen.bmj.com/site/about/guidelines.xhtml

For peer review only

REFERENCES

1. Blencowe H, Cousens S, Oestergaard MZ, Chou D, Moller AB, Narwal R, et al. National, regional, and worldwide estimates of preterm birth rates in the year 2010 with time trends since 1990 for selected countries: a systematic analysis and implications. Lancet 2012; 379:2162–72.
2. Blencowe H, Cousens S, Chou D, Oestergaard M, Say L, Moller AB, et al.. Born too soon: the global epidemiology of 15 million preterm births. Reprod Health. 2013;10 Suppl 1:S2.
3. Lawn JE, Davidge R, Paul VK, von Xylander S, de Graft Johnson J, et al. Born too soon: care for the preterm baby. Reprod Health. 2013;10 Suppl 1:S5
4. Kangaroo Mother Care: a practical guide. Department of Reproductive Health and Research, World Health Organization (2003). Available at: http://apps.who.int/iris/bitstream/10665/42587/1/9241590351.pdf
5. Conde-Agudelo A, Díaz-Rossello JL. Kangaroo mother care to reduce morbidity and mortality in low birth weight infants. Cochrane Database of Systematic Reviews 2016, Issue 8. Art. No.: CD002771.DOI: 10.1002/14651858.CD002771.pub4
6. Every newborn: an action plan to end preventable deaths. World Health Organization (2014). Available at: http://apps.who.int/iris/bitstream/10665/127938/1/9789241507448_eng.pdf?ua=1
7. Vesel L, Bergh AM, Kerber KJ, Valsangkar B, Mazia G, Moxon SG, et al. Kangaroo mother care: a multi-country analysis of health system bottlenecks and potential solutions. BMC Pregnancy Childbirth. 2015;15 Suppl 2:S5.
8. Bergh AM, Charpak N, Ezeonodo A, Udani R, van Rooyen E. Education and training in the implementation of kangaroo mother care. S Afr J Child Health. 2012;6(2):38–45.
9. The state of the world's children 2016: a fair chance for every child. United Nations Children's Fund (UNICEF), 2016. Available at: https://www.unicef.org/publications/files/UNICEF_SOWC_2016.pdf
10. Central Statistical Agency (CSA) [Ethiopia] and ICF. 2016. Ethiopia Demographic and Health Survey 2016: Key Indicators Report. Addis Ababa, Ethiopia,and Rockville, Maryland, USA. CSA and ICF. Available at: https://www.usaid.gov/sites/default/files/documents/1860/Ethiopia%20DHS%202016%20KIR%20-%20Final%2010-17-2016.pdf
11. The Last Ten Kilometers Project (L10K). Trends in reproductive, maternal, newborn and child health care practices in 115 L10K woredas: Analyses of three rounds of survey data. JSI Research & Training Institute, Inc./The Last Ten Kilometers (L10K) Project; 2015. Available at: http://l10k.jsi.com/Resources/Docs/L10KsRoundIIISurveyFinalReport.pdf
12. Federal Democratic Republic of Ethiopia Ministry of Health. HSTP Health Sector Transformation Plan 2015/16 – 2019/20 (2008–2012 EFY); 2015. Available at: https://www.globalfinancingfacility.org/sites/gff_new/files/Ethiopia-health-system-transformation-plan.pdf
13. Jackson R, Hailemariam A. The role of Health Extension Workers in linking pregnant women with health facilities for delivery in rural and pastoralist areas of Ethiopia. Ethiop J Health Sci. 2016;26(5):471-478.
14. Ethiopia- Population and Housing Census 2007; IPUMS subset. Available at: http://microdata.worldbank.org/index.php/catalog/2747

For peer review only - http://bmjopen.bmj.com/site/about/guidelines.xhtml

15. LSMS- Integrated surveys on agriculture; Ethiopia socioeconomic survey (ESS) 2015/2016. Central Statistical Agency and Living Standards Measurement Study (LSMS), World Bank.
16. Sankar MJ, Neogi SB, Sharma J, Chauhan M, Srivastava R, Prabhakar PK, et al. State of newborn health in India. J Perinatol. 2016; 36(Suppl 3): S3–S8.
17. Bhilwar M, Upadhyay RP, Yadav K, Kumar R, Chinnakali P, Sinha S et al. Estimating the burden of 'weighing less': A systematic review and meta-analysis of low birth-weight in India. Natl Med J India. 2016;29(2):73-81.
18. India Newborn Action Plan (INAP). Child Health Division, Ministry of Health and Family Welfare, Government of India. September 2014. Available at: https://www.newbornwhocc.org/INAP_Final.pdf
19. Kangaroo mother care and optimal feeding of low birth weight: Operational guidelines. Child Health Division. Ministry of Health and Family Welfare, Government of India. September 2014. Available at: http://nhm.gov.in/images/pdf/programmes/childhealth/guidelines/Operational_Guidelines-KMC_&_Optimal_feeding_of_Low_Birth_Weight_Infants.pdf
20. Census (2011). Office of the Registrar General and Census Commissioner. Ministry of Home Affairs, Government of India. Available at: http://www.censusindia.gov.in/2011census/PCA/PCA_Highlights/pca_highlights_file/India/Chapter-1.pdf
21. Census of India 2011. District Census Handbook, Sonipat. Directorate of Census Operations, Haryana. Available at: http://www.censusindia.gov.in/2011census/dchb/0608_PART_B_DCHB_SONIPAT.pdf
22. International Institute for Population Sciences (IIPS), 2014. District Level Household and Facility Survey (DLHS-4), 2012-13: India. Haryana; District fact sheet, Sonepat: Mumbai: IIPS. Available at: https://nrhm-mis.nic.in/DLHS4/State%20and%20District%20Factsheets/.../Sonipat.pdf
23. State Fact Sheet-Haryana; National Family Health Survey 2015-2016. Available at: http://rchiips.org/NFHS/pdf/NFHS4/HR_FactSheet.pdf
24. Sample registration system (SRS) Bulletin; Registrar General of India (2016). Available at: http://www.censusindia.gov.in/vital_statistics/SRS_Bulletin_2015.pdf
25. PHFI, AIIMS, and SC- State of India's Newborns (SOIN) 2014- a report. (Eds) Zodpey S and Paul VK. Public Health Foundation of India, All India Institute of Medical Sciences and Save the Children. New Delhi, India. Available at: https://www.newbornwhocc.org/SOIN_PRINTED%2014-9-2014.pdf
26. Mony PK, Jayanna K, Bhat S, Rao SV, Crockett M, Avery L, et al. Availability of emergency neonatal care in eight districts of Karnataka state, southern India: a cross-sectional study. *BMC Health Serv Res* 2015; 15(1): 461
27. Annual Health Survey fact sheet; Uttar Pradesh (2012-13). Vital Statistics Division. Office of the Registrar General and Census Commissioner, Ministry of Home Affairs, Government of India. Available at: http://www.censusindia.gov.in/vital_statistics/AHSBulletins/AHS_Factsheets_2012-13/FACTSHEET-UTTAR_PRADESH.pdf
28. Seidman G, Unnikrishnan S, Kenny E, Myslinski S, Cairns-Smith S, Mulligan B, et al. Barriers and enablers of Kangaroo Mother Care practice: a systematic review. *PLOS ONE* 2015, 10(5):e0125643.
29. Chan GJ, Labar AS, Wall S, Atun R. Kangaroo mother care: a systematic review of barriers and enablers. *Bulletin of the World Health Organization* 2016, 94(2):130-141J.

For peer review only - http://bmjopen.bmj.com/site/about/guidelines.xhtml

30. Smith ER, Bergelson I, Constantian S, Valsangkar B, Chan GJ. Barriers and enablers of health system adoption of kangaroo mother care: a systematic review of caregiver perspectives. BMC Pediatr 2017; 17: 35.

For peer review only

For peer review only - http://bmjopen.bmj.com/site/about/guidelines.xhtml

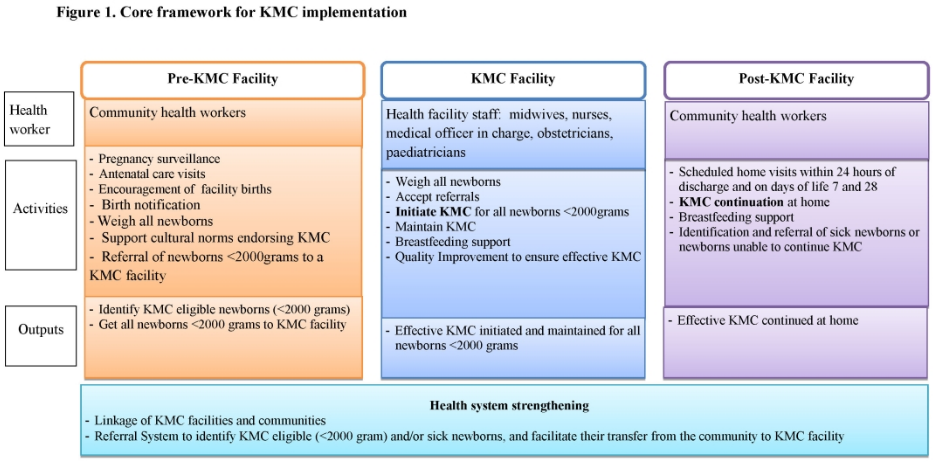

Figure 1. Core Framework for KMC Implementation

270x137mm (300 x 300 DPI)

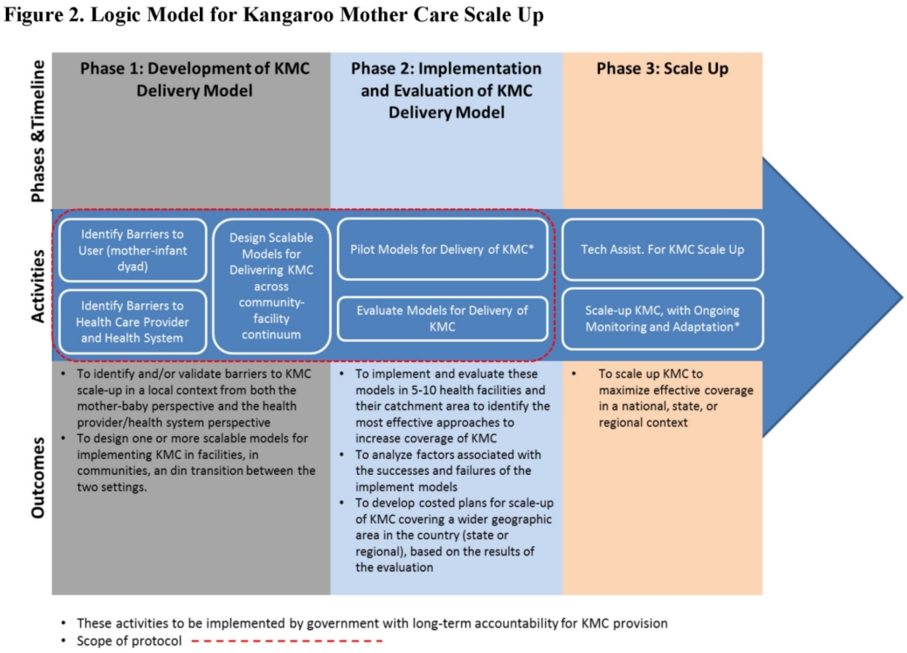

Figure 2. Logic Model for Kangaroo Mother Care Scale Up

198x140mm (300 x 300 DPI)

BMJ Open

Kangaroo Mother Care Implementation Research to Develop Models for Accelerating Scale-up in India and Ethiopia: Study Protocol for an Adequacy Evaluation

Journal:	*BMJ Open*
Manuscript ID	bmjopen-2018-025879.R1
Article Type:	Protocol
Date Submitted by the Author:	17-Jul-2019
Complete List of Authors:	Bahl, Rajiv; World Health Organization<br>Cranmer, John; Emory University, Nell Hodgson Woodruff School of Nursing<br>Gobezayehu, Abebe; Emory University Ethiopia<br>Chan, Grace; Harvard Medical School; Boston Children's Hospital , Division of Medicine Critical Care<br>Mariam, Damen; Addis Ababa University School of Public Health<br>Tadele, Henok; Hawassa University<br>Mazumder, Sarmila; Society for Applied Studies, Centre for Health Research and Development<br>Mony, Prem; St. John's Research Institute, St. John's National Academy of Health Sciences, Division of Epidemiology & Population Health<br>Kumar, Vishwajeet ; St. John's Medical College & Research Institute, Community Health and Epidemiology<br>Martines, Jose; Universitetet i Bergen Senter for internasjonal helse, Centre for Intervention Science in Maternal and Child Health<br>Pn Rao, Suman; St John's Medical College Hospital, Department of Neonatology<br>Darmstadt, G; Stanford University School of Medicine, Department of Pediatrics<br>Krishna, Raghav ; Community Empowerment Lab, Global Health<br>Kajal, FNU; Indian Administrative Service, National Health Mission<br>Pileggi-Castro, Cynthia; Centro de Referência da Saúde da Mulher de Ribeirão Preto, Maternidade<br>Sibley, Lynn M.; Emory University School of Public Health, Global Health<br>Jayanna, K; Karnataka Health Promotion Trust, Bangalore, Karnataka, India, Quality Improvement<br>Mohan, H L; Karnataka Health Promotion Trust, Community Mobilization<br>Kar, Arin; Karnataka Health Promotion Trust, Department of Health & Family Welfare<br>Amin, Hajira; Emory University , M&E<br>Upadhyay, Ravi; Centre for Health Research and Development,<br>Madhur, Tarun; Society for Applied Studies, Centre for Health Research and Development<br>Jadaun, Arun; Society for Applied Studies, Centre for Health Research and Development<br>Bhandari, Nita; Society for applied studies, Centre for Health Research and Development<br>Kumar, Aarti; Foundation for Community Empowerment, Global Health

	Estifanos, Abiy; Addis Ababa University School of Public Health<br>Fikre, Addisalem; Addis Ababa University School of Public Health<br>Alemu, Hilbret; John Snow Inc, Urban Health<br>Tariku, Abraham; Federal Ministry of Health<br>Duguma, Dereje; Oromia Regional Health Bureau<br>Medhanyie , Araya; Mekelle University College of Health Sciences, School of Public Health<br>Abay, Tedros; Mekelle University College of Health Sciences, Department of Pediatrics and Child Health<br>Beyene , Selemawit; Mekelle University College of Health Sciences, School of Public Health<br>Gebregizabher , Fisseha; Tigray Regional Health Bureau<br>Hadush, Marta; Mekelle University College of Health Sciences, Department of Pediatrics and Child Health<br>Lemango , Ephrem; Federal Ministry of Health , Maternal and Child Health Directorate<br>Tadesse, Birkneh; Hawassa University College of Medicine and Health Sciences, Department of Pediatrics and Child Health<br>Asefa, Anteneh; Hawassa University College of Medicine and Health Sciences, School of Public Health<br>Beyene, Habtamu; SNNPR Regional Health Bureau<br>Worku, Bogale; Pediatrics Society; Addis Ababa University<br>Muleta, Mekonnen; Private Consultant<br>Brune, Thomas; Karloniska Institute, Department of Neonatology<br>Singhal, Nalini; University of Calgary Cumming School of Medicine, Department of Neonatology<br>Aziz, Khalid; University of Alberta, Department of Neonatology<br>Belew, Mulusew; Emory University, Amhara Regional Office<br>Andualem, Bizuayehu; Amhara National Regional Health Bureau<br>M, Rajini; Government of Karnataka, Department of Health & Family Welfare

**Primary Subject Heading**:	Health services research
Secondary Subject Heading:	Paediatrics, Global health
Keywords:	kangaroo-mother care method, low birth weight, newborn, research protocol

SCHOLARONE™ Manuscripts

TITLE PAGE

KANGAROO MOTHER CARE IMPLEMENTATION RESEARCH TO DEVELOP MODELS FOR ACCELERATING SCALE-UP IN INDIA AND ETHIOPIA: STUDY PROTOCOL FOR AN ADEQUACY EVALUATION

KMC Scale-Up Study Group*

Corresponding author:

Rajiv Bahl – Coordinator MRD, Department of Maternal, Newborn, Child and Adolescent Health, World Health Organization. Geneva 1211, Switzerland (email: bahlr@who.int)

Phone number: +4179 4755492

Financial disclosure statement: The authors have no financial relationship relevant to this article to disclose

Funding / Support: The World Health Organization, the sponsor, will fund the study with financial support from the Bill and Melinda Gates Foundation. WHO facilitated the preparation of the protocol, will monitor implementation and analysis of data. It will also facilitate the writing of the report and participate in the decision to submit the report for publication. The study teams will have ultimate authority over any of these activities.

Potential conflicts of interest: The authors have no conflicts of interest relevant to this article to disclose.

Clinical Trial registry name and registration number:

Community Empowerment Laboratory, Uttar Pradesh, India - ISRCTN12286667

St John's National Academy of Health Sciences, Bangalore, India and Karnataka Health Promotion Trust, Bangalore, India - CTRI/2017/07/008988.

Society for Applied Studies, Delhi - NCT03098069

Oromia, Ethiopia - NCT03419416.

Amhara, SNNPR and Tigray, Ethiopia - NCT03506698.

For peer review only - http://bmjopen.bmj.com/site/about/guidelines.xhtml

List of abbreviations

CHW- community health worker

HEW- Health extension worker

KMC- Kangaroo mother care

LBW- Low birth weight

L10K- Last ten kilometers project

NICU- Neonatal intensive care unit

QA- Quality assurance

QI- Quality improvement

SNCU- Special neonatal care unit

SNNPR- Southern Nations Nationalities and People's Region

UP- Uttar Pradesh

WHO- World Health Organization

Short running title: Kangaroo Mother Care implementation research protocol

For peer review only

For peer review only - http://bmjopen.bmj.com/site/about/guidelines.xhtml

Contributor's statement page

All authors contributed to the preparation of the manuscript. The specific contributions are listed below:

Conceptualization of the study and first drafting of the manuscript: Abebe Gebremariam Gobezayehu, Mulusew Lijalem, Lyn Sibley, Grace J Chan, Abiy Seifu Estifano, Damen Haile Mariam, Henok Tadele, Birkneh Tilahun Tadesse, Khalid Aziz, Araya Abrha Medhanyie, Tedros Hailu Abaym, Sarmila Mazumder, Ravi Prakash Upadhyay, Nita Bhandari, Prem K. Mony, Suman Rao, Krishnamurthy Jayanna, Arin Kar, Aarti Kumar, Gary Lee Darmstadt, Vishwajeet Kumar, Rajiv Bahl, Cynthia Pileggi-Castro, Jose Carlos Martines

Subsequent revisions of manuscript drafts, completion of information on study settings and methods: John Cranmer, Abebe Gebremariam Gobezayehu, Mulusew Lijalem Belew, Bizuayehu Gashaw Andualem, Hajira Mohammed Amin, Lyn Sibley, Grace J Chan, Abiy Seifu Estifanos, Addisalem Fikre, Hibret Alemu, Abraham Tariku, Dereje Duguma, Damen Haile Mariam, Henok Tadele, Birkneh Tilahun Tadesse, Anteneh Asefa, Habtamu Beyene, Bogale Worku, Mekonnen Muleta, Thomas Brune, Nalini Singhal and Khalid Aziz, Araya Abrha Medhanyie, Tedros Hailu Abaym, Selemawit Asfaw Beyene, Fisseha Ashebir Gebregizabher, Marta Yemane Hadush, Ephrem Tekle Lemango, Sarmila Mazumder, Ravi Prakash Upadhyay, Tarun Kumar Madhur, Arun Jadaun, Nita Bhandari, Prem K. Mony, Suman Rao, Krishnamurthy Jayanna, Arin Kar, Mohan H. L., Rajini M., Aarti Kumar, Raghav Krishna, Kajal, Gary Lee Darmstadt, Vishwajeet Kumar, Rajiv Bahl, Jose Carlos Martines.

All authors read and approved the final manuscript.

Patients and public were not involved in the development of the protocol.

For peer review only

For peer review only - http://bmjopen.bmj.com/site/about/guidelines.xhtml

ABSTRACT

Introduction: Kangaroo Mother Care (KMC) is the practice of early, continuous and prolonged skin-to-skin contact between the mother and the baby with exclusive breastfeeding. Despite clear evidence of impact in improving survival and health outcomes among low birth weight infants, KMC coverage has remained low and implementation has been limited. Consequently, only a small fraction of newborns that could benefit from KMC receive it.

Methods and analysis: This implementation research project aims to develop and evaluate district-level models for scaling up KMC in India and Ethiopia that can achieve high population coverage. The project includes formative research to identify barriers and contextual factors that affect implementation and utilization of KMC and design scalable models to deliver KMC across the facility-community continuum. This will be followed by implementation and evaluation of these models in routine care settings, in an iterative fashion, with the aim of reaching a successful model for wider district, state and national-level scale-up. Implementation actions would happen at three levels: "pre-KMC facility" - to maximize the number of newborns getting to a facility that provides KMC; "KMC facility" - for initiation and maintenance of KMC; and "post-KMC facility" - for continuation of KMC at home. Stable infants with birth weight <2000 g and born in the catchment population of the study KMC facilities would form the eligible population. The primary outcome will be coverage of KMC in the preceding 24 hours and will be measured at discharge from the KMC facility and seven days after hospital discharge.

Ethics and dissemination: Ethics approval was obtained in all the project sites, and centrally by the Research Ethics Review Committee at the World Health Organization. Results of the project will be submitted to a peer-reviewed journal for publication, in addition to national and global level dissemination.

Strengths and limitations of the study

- This will be the first implementation research project to develop and evaluate models for achieving high population coverage with KMC.
- The project is a partnership between research groups and local governments, implemented at large scale, covering populations in diverse regions in two countries, Ethiopia and India, of high prevalence of low birth weight.

For peer review only - http://bmjopen.bmj.com/site/about/guidelines.xhtml

- The models' success in increasing KMC coverage will be assessed through the comparison with data at the beginning of the project that indicated that KMC is a rare practice in these settings, covering under 5% of low birth weights, and that competing intervention promoting KMC are not currently implemented in study sites.

For peer review only

For peer review only - http://bmjopen.bmj.com/site/about/guidelines.xhtml

INTRODUCTION

Globally, each year 15 million neonates are born preterm and consequently at a high risk of mortality.[1,2] South Asia and sub-Saharan Africa account for almost two-thirds of the world's preterm babies.[3] Kangaroo Mother Care (KMC) is a low-cost intervention involving early, continuous and prolonged skin-to-skin contact between mother and baby and exclusive breastfeeding.[4] KMC has been demonstrated to promote physiologic stability, a thermally-supportive environment, reduce risk of serious infections, and reduce the mortality among hospitalized, stable preterm and low birth weight (LBW) infants.[5] Despite the benefits, coverage of KMC has remained low and implementation has largely been limited to specialized hospitals.[6-8] As a result, only a small fraction of newborns that could benefit from KMC receive it. A set of seven studies has been planned in Ethiopia and India to develop and test models of scaling-up KMC effectively.

AIMS OF THE IMPLEMENTATION RESEARCH

Aim: To develop a delivery model that will result in high coverage (80%+) and quality of KMC for the target population.

Additional aims:

A: To design and identify the most promising delivery model through formative research combined with an iterative quality improvement (QI) approach.

B: To implement the most promising model in the study facilities and evaluate quality and coverage of KMC in the population they serve.

METHODS/ DESIGN

We are undertaking an international, multi-site, implementation study using a mixed-methods design that integrates formative and evaluative components into iterative improvement cycles. Our intervention promotes KMC for babies born <2000 g across the community-facility continuum.

Population

The population will be newborns with birth weight under 2000 g born in the geographic area targeted by the intervention. It includes all babies born in health facilities and in the community. For those

For peer review only - http://bmjopen.bmj.com/site/about/guidelines.xhtml

newborns who are severely sick according to pre-defined criteria (not tolerating oral feeds, severe respiratory distress including, respiratory rate less than 20 breaths per minute, grunting, central cyanosis, severe chest indrawing, convulsions, unconsciousness, and severe hypothermia of less than 32 degrees Celsius), KMC will be delayed until they stabilized.

Intervention

The intervention will be the provision of KMC, defined as skin-to-skin contact with the mother or a replacing caregiver, for as long as possible during day and night, with a minimum of at least eight hours over a 24-hour period, along with exclusive breastfeeding or breast milk feeding via tube, spoon or paladai. Based on the available evidence of effectiveness, KMC will only be initiated in health facilities.[5]

Comparison

The performance of the interventions will be assessed against a predefined success criterion of 80% or higher coverage of effective KMC at population level.

Outcomes

The assumption that current coverage with KMC initiated at a health facility is low or negligible in the study population will be confirmed by a baseline assessment of facilities.

The primary outcome will be effective coverage of KMC, defined as at least eight hours of skin-to-skin contact in combination with exclusive breastfeeding in the preceding 24 hours among infants with birth weight <2000 g. It will be measured:

- at discharge from the KMC facility (defined as one where staffing, infrastructure and mechanisms are present to initiate and maintain KMC)

- at 7 days after discharge, in the home

- at 28 days of age, in the home

Secondary outcomes include:

— Population-level duration of KMC, i.e. mean of how many days KMC was received by infants in the population with birth weight <2000 g during the neonatal period.

For peer review only - http://bmjopen.bmj.com/site/about/guidelines.xhtml

- Proportion of infants with birth weight <2000 g receiving any KMC (any duration in last 24 hours) at discharge, seven days post discharge and at 28 days of age
- Proportion of infants with birth weight <2000 g exclusively breastfed (based on 24-hour recall) at discharge, seven days post discharge and at 28 days of age
- Neonatal mortality rate (including early neonatal mortality rate) in infants <2000 g
- Cost of the KMC model (measured in some of the sites)

Sample Size

We assume that 3-5% of neonates born in Ethiopia and India will be <2000 g and that it will not be possible to evaluate 20% of these newborns because of early death or loss to follow up. A sample size of 310 newborns per site will allow us to estimate 80% coverage of KMC with site-specific absolute precision of ±5%. All the study sites will have sufficient numbers of births of eligible infants to enrol at least 310 during the evaluation phase. We anticipate the follow-up of a total of at least 2170 newborns <2000 g during the evaluation period.

Study Sites

The study will be conducted in a total of seven sites in Ethiopia and in India. Ethiopia and India are among the countries in Africa and Asia with the highest proportion of low birth weight babies. Selection of sites within each country considered the quality of the proposals and the desire to reflect regional diversity.

Ethiopia

In Ethiopia, newborn mortality remains high at 29 per 1000 live births.[9,10] The Ministry of Health has spearheaded a major campaign to increase facility births which appears effective.[11-15] Results from a recent survey (L10K) in 2015 conducted in 115 districts (woredas) in agrarian regions, indicate facility births in the past 12 months to be around 53%.[11]

Amhara

The study will be conducted in two woredas of West Gojam Zone, two woredas of South Gondar Zone, and Bahir Dar City Administration, Amhara Region. In West Gojam zone, the study includes one primary hospital. In Bahir Dar City Administration, the study includes a large referral hospital, one primary hospital, 19 health centres, 42 health posts and their respective catchment populations. In South Gondar Zone, the study includes one general hospital and one primary

For peer review only - http://bmjopen.bmj.com/site/about/guidelines.xhtml

hospital, 14 health centres and up to 59 health posts affiliated with these health centres, along with their respective catchment populations.

Oromia – Addis Ababa

The Oromia – Addis Ababa site will implement the study in Akaki Kality sub-city in Addis Ababa and East Shoa zone in Oromia region. Akaki Kality sub-city, one of the 10 sub-cities of Addis Ababa city administration shares border and culture with East Shoa zone of Oromia region. The Akaki Kality sub-city has an estimated population of 180,000 living in 11 woredas in the sub-city.[14] Oromia Regional state has an estimated population of 27 million [14]. The study will be implemented in two woredas: Adami Tulu in the East Shewa Zone and Arsi Robe in the Arsi Zone.

SNNPR

SNNPR has a facility birth rate of 25.2%.[10] The sites proposed for study are Hawassa city administration, Shebedino woreda and Dale woreda, situated in Sidama zone. Hawassa is the capital of SNNPR and its administration consists of eight sub-cities with urban and rural kebeles. The two public hospitals in Hawassa city—namely Hawassa University Comprehensive Specialized Hospital and Adare General Hospital—are included in the study. Leku is the capital of Shebedino woreda and consists of urban and rural kebeles. Leku Primary Hospital is the site for Shebedino woreda. Yirgalem is the capital of Dale woreda. Yirgalem General Hospital is the site for Dale woreda.

Tigray

According to the EDHS 2016, in the Tigray region, 69% of the women who gave birth two years preceding the survey delivered in facilities. The study will be implemented in two zones, namely south-east zone and Mekelle special zone. In the south-east zone, two districts (Enderta and Degu-Tembien woredas) will be included in the study. In the selected districts and Mekelle Zone, there are one comprehensive referral hospital, two general hospitals, one primary hospital, 22 health centres and five private health facilities. All these health facilities will be included in the study

For peer review only - http://bmjopen.bmj.com/site/about/guidelines.xhtml

India

India accounts for one quarter of newborn deaths in the world. Neonatal deaths represent about 50% of under-five deaths and 70% of its infant deaths.[16] About 27% of all babies are low birth weight (<2500g).[17-20]

Haryana

The study will be implemented in one of Haryana's 22 districts (Sonepat). The low birth weight rate is 18% in Sonepat.[21,22] Around 5% of all infants weigh less than 2000 g at birth (*information from government records*). The institutional delivery rate is 80.5% and 90% of births are conducted by skilled birth attendants.[23] Infant mortality in Haryana is 36/1000, similar to the Indian average.[24] The neonatal mortality rate (NMR) is 28/1000, a major proportion of infant mortality rate.[25]

Karnataka

Infant mortality rate is 28/1000 and is NMR is 22/1000 live births.[24,25] The research project will be conducted in the Koppal district, located 400 km away from Bangalore. This is an under-served, in the northern region of the state identified as a high priority district by the government.[26] The district has a low birth weight prevalence of about 25% and a NMR of 42/1000. About 80% of births are institutional deliveries.

Uttar Pradesh (UP)

UP is the most populous state in India and accounts for a quarter of India's burden of neonatal deaths.[25] UP's NMR of 37/1000 is amongst the highest in the country.[25] The prevalence of low birth weight in UP is 25%.[27] The study will be implemented in the Raebareli district with an institutional delivery rate of 93%. The study will cover the district hospital and ten community health centres, along with their catchment blocks.

Ethical Considerations

Since the intervention will be provided by the government as standard of care for LBW, there will be no request for consent from individuals to receive the intervention. Individual written informed consent will be requested from mothers, caregivers, and health workers for the collection of information. For those unable to read, the informed consent form will be read out by the research team member before asking consent.

Patient and Public Involvement

For peer review only - http://bmjopen.bmj.com/site/about/guidelines.xhtml

The question addressed by this research project was identified as a priority by the governments of Ethiopia and India. They participated in planning the study through the ministries of health. Patients and communities will play a major role in designing the models developed and tested, as described below under Phase 1 – Formative research. Results will be disseminated to the communities using channels available to the health system, as well as to the state and national health authorities, and through publication in peer-reviewed journals.

Study approvals

Study approvals were obtained at the local, national and international levels. Ethics approvals were taken from the ethics committees of the institutions engaged in the project and centrally from the World Health Organization (WHO) Ethics Committee.

Study implementation strategy

Core Framework for KMC Implementation

KMC promotion will be implemented at three levels (Figure 1):

INSERT FIGURE 1 HERE

"Pre-KMC facility" - the aim at this level is to maximize the proportion of newborns under 2000 g to be born in/brought to KMC implementing health facilities.

"KMC facility" – the aim at this level is early initiation of KMC and adherence to long duration (>8 hours) skin-to-skin care with exclusive breastfeeding within the facility.

"Post-KMC facility" – the aim at this level is continuation of effective KMC at home through the neonatal period or until the baby no longer accepts it.

Study Teams

The KMC intervention will be delivered by health workers who are part of the health system. The research team's implementation role will be only supportive. This research team will be comprised of three small teams:

(i) The "program learning team", to conduct formative research and process-monitoring, help formulate the intervention and process-evaluation of implementation.

For peer review only - http://bmjopen.bmj.com/site/about/guidelines.xhtml

(ii) The “implementation support team” to guide and support the health system so that the environment supports KMC and health workers implementing the intervention.

(iii) The “outcome evaluation team” to measure the study outcomes in the population acting independently from the other two teams (Table 1).

For peer review only

For peer review only - http://bmjopen.bmj.com/site/about/guidelines.xhtml

Table 1. Description of Study Implementation Teams

	Research			Government Implementation
	Program learning team	Implementation support team	Outcome evaluation team	Health system team
Phase 1	Conduct formative research to develop the initial KMC delivery model.<br><br>Conduct program learning to understand how well the KMC delivery model is working and to redesign the model based on lessons learned.	Support government implementation of KMC.	In the catchment area of the facilities involved in Phase 1, collect quantitative data on:<br>• Births in facilities and community<br>• Births <2000 g in facilities and community<br>• Number of newborns receiving KMC at time of discharge from health facility, seven days post discharge and at 28 days of age<br>• Duration of skin-to-skin care	Deliver and monitor the KMC intervention.

For peer review only - http://bmjopen.bmj.com/site/about/guidelines.xhtml

Phase 2	Monitor process indicators to learn how implementation of KMC delivery models happens in all study facilities and identify ways to refine the model based on context-specific findings.	Assume an advisory role to support government implementation of KMC.	In the entire study population, collect quantitative data as above.	Deliver and monitor the KMC intervention.

For peer review only

For peer review only - http://bmjopen.bmj.com/site/about/guidelines.xhtml

Phases of implementation

The implementation will happen in two phases (Figure 2).

INSERT FIGURE 2 HERE

Phase 1: To Develop a Delivery Model for KMC

KMC Delivery Model Cycles of Improvement

The initial delivery model will be developed based on the formative research and synthesis of literature. Program learning from the delivery of this model and outcome measurements will immediately feed into a stage of model review and redesign/refinement, aimed at improving the coverage. We expect that at least three cycles of model development-implementation-evaluation-refinement will be needed until reaching a scalable high coverage model. Each cycle may take three to four months. The first cycle may involve one facility but the second and third will be larger, including up to one-third of all study facilities.

At the end of each cycle, findings will be shared with an advisory group of stakeholders and their input will be sought to improve the model. New iterations of the model will be developed and further refined and reviewed with advisors. Figure 3 presents this iterative implementation-optimization of the model.

INSERT FIGURE 3 HERE

Formative research

The study's first step will be the program learning research team undertaking formative research to identify and understand barriers and enablers to KMC in the specific context. We will follow the COM-B model to guide understanding of behaviour change in the implementation context, and the development of behavioural targets as a basis for designing interventions with users[28]. It proposes that people need capability (C: psychological or physical ability to enact a behaviour), opportunity (O: physical and social environment that enable a behaviour) and motivation (M: reflective and automatic mechanisms that activate or inhibit a behaviour) to perform a behaviour (B). Domains of the formative research are presented in Table 2. Based on the knowledge gathered, we will develop an initial model of KMC implementation at the study site.

For peer review only - http://bmjopen.bmj.com/site/about/guidelines.xhtml

Implementation of KMC test models

The initial model will be implemented in collaboration with facility and community workers with guidance from the implementation support team. Selection of facilities to implement KMC test models will be based on factors such as number of births, type of health workers, services delivered (e.g. presence of newborn intensive care units or special care nurseries) so that they capture a major proportion of deliveries in the study district. Implementation will occur at three levels: pre-KMC facility, KMC facility, and post-KMC facility (Figure 1).

For peer review only

For peer review only - http://bmjopen.bmj.com/site/about/guidelines.xhtml

Table 2. Domains of Enquiry for Formative Research

Domains of enquiry	Activities
**KMC Intervention** – to inform the design of the technical package of KMC	• Review KMC scientific literature; existing government guidelines on KMC; experience on implementation of KMC<br>• Develop guidelines and protocols for health staff in facility and community health care workers<br>• Develop communication materials for communities, pregnant women and their families
**Users and implementers** – to understand the barriers and enablers to KMC adoption and adherence	**Mothers/Families**<br>• Explore perceptions of mothers and family members around preterm babies, low birth weight, care-seeking, institutional deliveries, private facilities, perception of quality; understand and explore ideas to generate demand for KMC<br>**Health Workers**<br>• Assess understanding and experience of KMC; understanding of birth weight, prematurity<br>• Assess knowledge, skill, time and motivation for newborn care
**Health systems** – to improve the delivery of KMC at the facility and community level	**Health facilities**<br>• Understand current KMC practices in health facilities; map behaviours that could impact adoption of KMC within these facilities<br>• Design a KMC facility readiness checklist based on local feasibility and provisions<br>• Assess physical infrastructure and resources available for KMC<br>**Link between community and facility**<br>• Assess current engagement between CHWs and health facilities and explore efficient process of engagement<br>• Assess referral system<br>**Assess data needs, measurement systems and analytics support and its compatibility and adaptability for KMC**<br>• Assess existing management information system; assess registries that exist at each level of the health care system<br>• Design and test electronic data management systems to guide and accelerate adoption and improvement of KMC<br>**Leadership and policy makers**

For peer review only - http://bmjopen.bmj.com/site/about/guidelines.xhtml

	• Engage leadership and decision makers for their support of KMC<br>• Assess need of additional workers and current human resource capacity gap<br>**Other stakeholders**<br>• Map and engage all professional, organizational, health system, administrative and social networks that may impede or support KMC adoption and spread in the study area
**Context** – to develop adaptable delivery models	• Understand perceptions of care of small babies and logistical challenges to care of high risk babies within the setting<br>• Explore motivations for facility versus home deliveries<br>• Map relationships between doctors and nurses, CHWs, and mothers/families

For peer review only

For peer review only - http://bmjopen.bmj.com/site/about/guidelines.xhtml

Pre-KMC facility

To effectively implement KMC, eligible newborns need to be identified and cared for in facilities where KMC is initiated. All births should be identified, and babies weighed. Eligible babies, if born outside KMC facilities, will be referred to these facilities through strengthened government transport and linkage mechanisms. The pre-facility level includes efforts to: increase facility deliveries, expand early assessment of those delivered at home, so that eligible newborns are referred to a KMC facility, and create a social norm around the practice of KMC.

KMC facility

Facility level activities will aim to strengthen knowledge and skills of health providers and implement KMC protocols so that they provide high-quality care. All stable newborns would be placed in skin-to-skin contact and initiated breastfeeding within the first hour of life. In-born and referred newborns would be screened for KMC eligibility (< 2000 g and stable). If meeting eligibility criteria, KMC would be initiated. If the newborn is sick, he/she will be stabilized first and then reassessed for KMC eligibility and initiation.

During the newborn's facility stay, nurses will do a KMC assessment following a checklist which examines skin-to-skin contact (number of hours) and breastfeeding, counselling on KMC, and problem-solving if barriers exist. Nurses will document the adoption of KMC. At time of discharge, mothers and family members will be counselled so that KMC is continued at home. Facility staff will notify community health workers (CHWs) about discharges and discuss how to support continued KMC at home.

Post-KMC facility

After discharge, CHWs shall visit the home within 24 hours following the post-facility KMC protocol. CHWs will continue to visit the newborn following the standard national postnatal care visit schedule. At each visit, CHWs will perform a KMC assessment using the follow-up checklist including skin-to-skin duration and breastfeeding, counselling and problem solving. CHWs will identify and refer sick newborns to the facility and assist the referral.

Health systems strengthening

Linkages between facility and community will be strengthened so that KMC is continued at home. This will be facilitated through establishment of communication systems between facilities and CHWs.

For peer review only - http://bmjopen.bmj.com/site/about/guidelines.xhtml

Outcome Measurement

The study outcome evaluation team will conduct an independent evaluation of the effectiveness of the model in achieving 80% or higher coverage with KMC among the target population. Data will be collected at health facilities and the community.

Phase 2: Implement and Evaluate the chosen KMC Delivery Model

The most promising KMC delivery model will be chosen from Phase 1 based on program learning findings and coverage. During Phase 2, the chosen model will be implemented in all selected facilities and data will be collected in the study area to measure the coverage with effective KMC.

Implementation of the KMC Delivery Model

The model will include KMC guidelines, protocols, communication and training materials, and tools for successful implementation. The delivery model will be implemented by the health system in all study facilities and their catchment areas. The implementation support team will continue to be active but now playing only an advisory role to the government implementation teams. There will be ongoing mentorship and quality improvement feedback cycles within each facility.

Implementation activities will include guideline dissemination, health care provider and CHW training, identification of low birth weights, community awareness and demand generation, establishment of referral and supervision systems, monitoring, and continuous feedback loops. Key activities are further described below.

Community awareness and demand generation: Contacts between families the health services will be utilized to promote awareness of KMC and of KMC units. Pregnant women and mothers of under-fives, for example, come in contact with health services during antenatal care, immunization sessions and routine home visits by CHWs. Additional activities, such as village health promotion days, will be used. Messages developed will be communicated through posters, pamphlets, banners and wall paintings and disseminated at strategic locations in the community and at facilities with maternity wards, antenatal clinics, postnatal clinics, paediatrics wards in addition to the KMC wards. Audiovisual media such as local network for television channels or local radio may also be used.

Consistent messages from multiple channels including interpersonal communication with health providers in public and private sectors and CHWs, will reinforce the information. Experience indicates that the endorsement from doctors seems critical to adoption of KMC. Orientation sessions will be conducted with all stakeholders and community representatives likely to influence community behaviour and norms.

For peer review only - http://bmjopen.bmj.com/site/about/guidelines.xhtml

Identification of resources and staffing within the health system: KMC is a new intervention for most health facilities in Ethiopia and India. Before initiating field work, discussions with national and local government officials will be held to promote buy-in. During these meetings, a landscaping overview of staffing, equipment and supplies at study health facilities will be completed. Together with government partners, research teams will meet with the leadership at study health facilities to introduce the KMC initiative, discuss their concerns and engage their involvement. We will jointly identify staff to participate in KMC implementation at each level of the health system.

Development of intervention materials: With Ministries of Health and country-specific teams, we will develop a standardized set of KMC protocols, guidelines, training materials, job tools, and checklists to support implementation. Existing training manuals on newborn care in Ethiopia and India include KMC training. We will update them to include strategies to initiate and continue KMC at health facilities and plans for follow-up care at home. These study manuals will be used to train care providers in the selected study health facilities. To support health workers in implementing KMC, we will develop job-aids, checklists and data collection forms.

Launch of the KMC implementation program: A one-day workshop will be conducted involving officials and experts from study sites including regional and district health offices, health facilities, and MNCH implementing partners. The workshop will orient stakeholders and strengthen local partnerships for successful implementation of project activities.

Implementation team training: We will provide an intensive training on KMC for health care workers from study facilities and CHWs from the community. Training will involve lectures and hands-on training, including technical updates, counselling skills, follow up care at health facility and at home, and documentation of the KMC services. Trainers will be experienced local paediatricians and faculty from the site-specific institutions and the pool of trainers already available in the state. Training components will include advantages of KMC, appropriate KMC techniques, possible barriers and ways to address them, how to support mothers to initiate and sustain KMC and counselling mothers/family members at discharge. Practical training will be coordinated centrally, at a dedicated KMC unit, initially through simulators and subsequently with mothers and their babies. Refresher training sessions will be held. Follow up with supportive supervision will be set up to ensure that the physicians and nurses translate their learning into implementation at their facilities. The supportive supervision will be initially ensured by the study team.

For peer review only

For peer review only - http://bmjopen.bmj.com/site/about/guidelines.xhtml

The CHWs and their supervisors will be trained on essential newborn care. Master trainers will be involved. The monthly meetings of the CHWs will provide opportunities for follow up re-training and query-solving of problems encountered during their work in the community.

Immediately after training, Objective Structured Clinical Examinations (OSCEs) will be conducted to test trainees' knowledge and skills. Mentors' training will be done at each level of the health system.

Process-monitoring

The program learning team will transition to documenting implementation processes by measuring process indicators. Process indicators for KMC implementation were described in Table 3.

For peer review only

For peer review only - http://bmjopen.bmj.com/site/about/guidelines.xhtml

Table 3. Process Indicators for KMC Implementation

Process	Indicator
Identification of pregnant women	• Proportion of pregnant women identified by CHW in the antenatal period
Exposure to KMC priming interactions in the antenatal period	• Proportion of identified pregnant women who received KMC information in the antenatal period from CHWs (either at home visits or during planned community interactions<br>• Proportion of identified pregnant women who received KMC information during ANC visits at health facilities
Delivery in KMC facility	• Proportion of women who deliver in a KMC facility versus in another facility or at home
Weighed at birth	• Proportion of live born babies who were weighed at birth, overall and by place of birth
Identification of newborns <2000 g	• Proportion of babies identified as <2000 g, overall and by place of birth
Referral	• Proportion of newborns <2000 g who were born at home or in non-KMC facility who were successfully referred to KMC facility for care
Initiation of KMC	• Proportion of newborns <2000 g born in KMC facility who were initiated on KMC<br>• Proportion of newborns <2000 g referred to KMC facility who were initiated on KMC
KMC monitoring	• Proportion of KMC initiated newborns monitored by facility staff according to protocol<br>• Proportion of facility KMC newborns discharged according to criteria; left against medical advice; referred out; died before discharge<br>• Duration of stay in facility
KMC follow-up	• Proportion of newborns discharged from facility receiving KMC who received follow-up per protocol

For peer review only - http://bmjopen.bmj.com/site/about/guidelines.xhtml

Outcome Measurement

The outcome measurement team will evaluate the effectiveness of the model in achieving 80% or higher coverage with KMC among the entire study population as described above in the outcome measurement section.

Evaluation

Effective KMC coverage is defined as the number of newborns receiving KMC divided by the total number of newborns eligible for KMC (<2000 g and stable) in the facilities and in the study population during the evaluation period.

To measure the denominator for effective KMC coverage it is preferred that all facility births be identified by the outcome measurement team during facility visits, review of birth registers, and/or calls to the facilities. In addition, the outcome measurement team will liaise with CHWs to obtain the number of home births and their birth weights.

To calculate the numerator for effective KMC coverage, the outcome measurement team will quantify the number of newborns under 2000 g who received KMC in the study population. All identified newborns under 2000 g will be followed by the outcome measurement team in the facility or at home to collect data at four points i.e. day of initiation of KMC, day of discharge from the KMC facility, seven days post-discharge, and at age 28 days. At each time point, the team will measure KMC based on records, and/or maternal report, if post-discharge.

Timeline

The study time-line is displayed in Figure 4.

INSERT FIGURE 4 HERE

Data Management

Data collected will be linked to participant identification numbers. Each research team will be responsible for data management for its site. The cleaned data set will be sent to WHO at monthly intervals for review and feedback. Data quality assurance methods would include online real-time checks for missing values and inconsistency; random spot checks and data validation will be coupled with independent quarterly audits.

For peer review only - http://bmjopen.bmj.com/site/about/guidelines.xhtml

Oversight

The Maternal, Newborn, Child and Adolescent Health (MCA) Department of WHO, Geneva will create a Steering Committee and be responsible for organizing its meetings. The Steering Committee will be the technical body responsible for the oversight.

DISCUSSION

Despite its scientifically documented benefits, scaling up of KMC has proven an elusive goal for about 40 years. With increased awareness of the magnitude of newborn mortality and the clear interest of the governments of Ethiopia and India in making KMC available to all newborns of birth weight below 2000 g, it is timely to implement this project. It provides a great opportunity to weave KMC within the existing health system and, in collaboration with the government, to find effective models of implementation applicable at scale and sustainable. The findings from this implementation research project will provide inputs to policy-makers to formulate KMC implementation guidance for state or nationwide scale up, if one doesn't yet exist, or to improve existing ones. The findings will have implications for additional actions to strengthen provision of essential newborn care.

Literature on barriers and facilitators of KMC within health facilities is available.[29-31] This project aims to develop models and test them in cycles with an inbuilt iterative learning component and strong government-research partnerships, that will ultimately lead to identification of effective models of scale up in large country-wide settings. While we focus on KMC coverage in our outcomes, our aim is to develop a model that incorporates KMC as an integral component of newborn care so that the KMC scale up is not projected as a vertical program. The experiences from this research will not only inform us on the challenges to scale up KMC and ways to effectively address them but will have the potential to help the design and implementation strategy of future infant and child care programs in a way that their accelerated uptake by health system and the communities is strengthened. The findings of this implementation research will also be useful in other low- and middle-income countries, besides India and Ethiopia.

For peer review only - http://bmjopen.bmj.com/site/about/guidelines.xhtml

***KMC Scale-Up Study Group**

Group authorship by region/state of implementation

ETHIOPIA

Amhara Region –

Principal Investigator: John Cranmer, Emory University, Nell Hodgson Woodruff School of Nursing Atlanta, GA, USA

Principal Investigator: Abebe Gebremariam Gobezayehu, Emory University Ethiopia, Addis Ababa, ET

Mulusew Lijalem Belew, Emory University, Amhara regional office, Bahir Dar, Ethiopia

Bizuayehu Gashaw Andualem, Amhara Regional Health Bureau, Bahir Dar, Ethiopia

Hajira Mohammed Amin, Emory University Ethiopia Country Office, Addis Ababa, Ethiopia

Lyn Sibley, Emory University, Atlanta, Georgia, USA

Oromia Region –

Principal Investigator: Grace J Chan, Harvard Medical School Boston, MA, USA; Boston Children's Hospital, Division of Medicine Critical Care, Boston, MA, USA

Principal Investigator: Damen Haile Mariam, Addis Ababa University, Addis Ababa, Oromia, ET

Abiy Seifu Estifanos, School of Public Health, Addis Ababa University, Addis Ababa, Ethiopia

Addisalem Fikre, School of Public Health, Addis Ababa University, Addis Ababa, Ethiopia

Hibret Alemu, JSI Urban Health, Addis Ababa, Ethiopia

Abraham Tariku, Federal Ministry of Health, Addis Ababa, Ethiopia

Dereje Duguma, Oromia Regional Health Bureau, Addis Ababa, Ethiopia

SNNPR (Southern Nations Nationalities and Peoples' Region) –

Principal Investigator: Henok Tadele, Hawassa University, Hawassa, Southern Nations, ET

Birkneh Tilahun Tadesse, Hawassa University, Department of Pediatrics and Child Health, Hawassa, Ethiopia

For peer review only - http://bmjopen.bmj.com/site/about/guidelines.xhtml

Anteneh Asefa, Hawassa University, School of Public Health, Hawassa, Ethiopia

Habtamu Beyene, SNNPR Regional Health Bureau, Hawassa, Ethiopia

Bogale Worku, Ethiopian Pediatrics Society and Addis Ababa University, Addis Ababa, Ethiopia

Mekonnen Muleta, Private Consultant, Addis Ababa, Ethiopia

Thomas Brune, Karloniska Institute, Department of Neonatology, Stockholm, Sweden

Nalini Singhal, University of Calgary, Department of Neonatology, Calgary, Canada

Khalid Aziz, University of Alberta, Department of Neonatology, Edmonton, Canada

Tigray Region –

Principal Investigator: Araya Abrha Medhanyie, Mekelle University, Mekelle, Tigray, ET

Tedros Hailu Abaym, Department of Pediatrics and Child Health, Mekelle University, Mekelle, Ethiopia

Selemawit Asfaw Beyene, School of Public Health, Mekelle University, Mekelle, Ethiopia

Fisseha Ashebir Gebregizabher, Tigray Regional Health Bureau, Mekelle, Ethiopia

Marta Yemane Hadush, Department of Pediatrics and Child Health, Mekelle University, Mekelle, Ethiopia

Ephrem Tekle Lemango, Maternal and Child Health Directorate, Federal Ministry of Health, Addis Ababa, Ethiopia

INDIA

Haryana –

Principal Investigator: Sarmila Mazumder, Centre for Health Research and Development, Society for Applied Studies, New Delhi, Delhi, IN

Ravi Prakash Upadhyay, Centre for Health Research and Development, Society for Applied Studies, New Delhi, India

Tarun Kumar Madhur, Centre for Health Research and Development, Society for Applied Studies, New Delhi, India

Arun Jadaun, Centre for Health Research and Development, Society for Applied Studies, New Delhi, India

For peer review only - http://bmjopen.bmj.com/site/about/guidelines.xhtml

Nita Bhandari, Centre for Health Research and Development, Society for Applied Studies, New Delhi, India

Karnataka –

Principal Investigator: Prem K. Mony, St John's Research Institute, Division of Epidemiology, Biostatistics & Population Health, Bangalore, IN

Suman Rao, St John’s Medical College Hospital, Department of Neonatology, Bangalore, IN

Krishnamurthy Jayanna, Karnataka Health Promotion Trust, Bangalore, Karnataka, India, Quality Improvement, Bangalore, IN

Arin Kar, Karnataka Health Promotion Trust, Bangalore

Mohan H. L., Karnataka Health Promotion Trust, Community Mobilization Bangalore, IN

Rajini, M. Department of Health & Family Welfare, Government of Karnataka, Bangalore

Uttar Pradesh –

Principal *Investigator: Vishwajeet Kumar,* Community Empowerment Lab, Global Health, Lucknow, India

Aarti Kumar, Community Empowerment Lab, Global Health, Lucknow, India

Raghav Krishna, Community Empowerment Lab, Global Health, Lucknow, IN

Kajal (no last name), Indian Administrative Service, National Health Mission, Lucknow, IN

Gary Lee Darmstadt, Stanford University School of Medicine, Department of Pediatrics, Stanford, CA, USA

World Health Organization

Principal Investigator: Rajiv Bahl, World Health Organization

20 Avenue Appia, World Health Organization, Geneva, CH

Cynthia Pileggi-Castro, Centro de Referência da Saúde da Mulher de Ribeirão Preto, Maternidade, Ribeirão Preto (SP), BR

Jose Carlos Martines, Universitetet i Bergen Senter for internasjonal helse, Centre for Intervention Science in Maternal and Child Health, Bergen, NO

Anayda Portela, World Health Organization, Department of Maternal, Newborn, Child and Adolescent Health, Geneva, CH

For peer review only - http://bmjopen.bmj.com/site/about/guidelines.xhtml

Study status

WHO approved protocol: Version 4 - 12 May 2016– Protocol ID: ERC 2716

Study implementation beginning: April 2017

Study end: expected March 2019

Acknowledgements

We acknowledge the editorial support of Daphne McRae and Bertrand Leduc in the preparation of the final manuscript.

Disclaimer

The authors alone are responsible for the views expressed in this [article][chapter] and they do not necessarily represent the views, decisions or policies of the institutions with which they are affiliated.

For peer review only

For peer review only - http://bmjopen.bmj.com/site/about/guidelines.xhtml

REFERENCES

1. Blencowe H, Cousens S, Oestergaard MZ, Chou D, Moller AB, Narwal R, et al. National, regional, and worldwide estimates of preterm birth rates in the year 2010 with time trends since 1990 for selected countries: a systematic analysis and implications. Lancet. 2012; 379:2162–72.
2. Blencowe H, Cousens S, Chou D, Oestergaard M, Say L, Moller AB, et al. Born too soon: the global epidemiology of 15 million preterm births. Reprod Health. 2013;10 Suppl 1:S2.
3. Lawn JE, Davidge R, Paul VK, von Xylander S, de Graft Johnson J, et al. Born too soon: care for the preterm baby. Reprod Health. 2013;10 Suppl 1:S5
4. Kangaroo Mother Care: a practical guide. Department of Reproductive Health and Research, World Health Organization (2003). Available at: http://apps.who.int/iris/bitstream/10665/42587/1/9241590351.pdf
5. Conde-Agudelo A, Díaz-Rossello JL. Kangaroo mother care to reduce morbidity and mortality in low birth weight infants. Cochrane Database of Systematic Reviews 2016, Issue 8. Art. No.: CD002771.DOI: 10.1002/14651858.CD002771.pub4
6. Every newborn: an action plan to end preventable deaths. World Health Organization (2014). Available at: http://apps.who.int/iris/bitstream/10665/127938/1/9789241507448_eng.pdf?ua=1
7. Vesel L, Bergh AM, Kerber KJ, Valsangkar B, Mazia G, Moxon SG, et al. Kangaroo mother care: a multi-country analysis of health system bottlenecks and potential solutions. BMC Pregnancy Childbirth. 2015;15 Suppl 2:S5.
8. Bergh AM, Charpak N, Ezeonodo A, Udani R, van Rooyen E. Education and training in the implementation of kangaroo mother care. S Afr J Child Health. 2012;6(2):38–45.
9. The state of the world's children 2016: a fair chance for every child. United Nations Children's Fund (UNICEF), 2016. Available at: https://www.unicef.org/publications/files/UNICEF_SOWC_2016.pdf
10. Central Statistical Agency (CSA) [Ethiopia] and ICF. 2016. Ethiopia Demographic and Health Survey 2016: Key Indicators Report. Addis Ababa, Ethiopia,and Rockville, Maryland, USA. CSA and ICF. Available at: https://www.usaid.gov/sites/default/files/documents/1860/Ethiopia%20DHS%202016%20KIR%20-%20Final%2010-17-2016.pdf
11. The Last Ten Kilometers Project (L10K). Trends in reproductive, maternal, newborn and child health care practices in 115 L10K woredas: Analyses of three rounds of survey data. JSI Research & Training Institute, Inc./The Last Ten Kilometers (L10K) Project; 2015. Available at: http://l10k.jsi.com/Resources/Docs/L10KsRoundIIISurveyFinalReport.pdf
12. Federal Democratic Republic of Ethiopia Ministry of Health. HSTP Health Sector Transformation Plan 2015/16 – 2019/20 (2008–2012 EFY); 2015. Available at: https://www.globalfinancingfacility.org/sites/gff_new/files/Ethiopia-health-system-transformation-plan.pdf
13. Jackson R, Hailemariam A. The role of Health Extension Workers in linking pregnant women with health facilities for delivery in rural and pastoralist areas of Ethiopia. Ethiop J Health Sci. 2016;26(5):471-478.
14. Ethiopia- Population and Housing Census 2007; IPUMS subset. Available at: http://microdata.worldbank.org/index.php/catalog/2747

For peer review only - http://bmjopen.bmj.com/site/about/guidelines.xhtml

15. LSMS- Integrated surveys on agriculture; Ethiopia socioeconomic survey (ESS) 2015/2016. Central Statistical Agency and Living Standards Measurement Study (LSMS), World Bank.
16. Sankar MJ, Neogi SB, Sharma J, Chauhan M, Srivastava R, Prabhakar PK, et al. State of newborn health in India. J Perinatol. 2016; 36(Suppl 3): S3–S8.
17. Bhilwar M, Upadhyay RP, Yadav K, Kumar R, Chinnakali P, Sinha S et al. Estimating the burden of 'weighing less': A systematic review and meta-analysis of low birth-weight in India. Natl Med J India. 2016;29(2):73-81.
18. India Newborn Action Plan (INAP). Child Health Division, Ministry of Health and Family Welfare, Government of India. September 2014. Available at: https://www.newbornwhocc.org/INAP_Final.pdf
19. Kangaroo mother care and optimal feeding of low birth weight: Operational guidelines. Child Health Division. Ministry of Health and Family Welfare, Government of India. September 2014. Available at: http://nhm.gov.in/images/pdf/programmes/childhealth/guidelines/Operational_Guidelines-KMC_&_Optimal_feeding_of_Low_Birth_Weight_Infants.pdf
20. Census (2011). Office of the Registrar General and Census Commissioner. Ministry of Home Affairs, Government of India. Available at: http://www.censusindia.gov.in/2011census/PCA/PCA_Highlights/pca_highlights_file/India/Chapter-1.pdf
21. Census of India 2011. District Census Handbook, Sonipat. Directorate of Census Operations, Haryana. Available at: http://www.censusindia.gov.in/2011census/dchb/0608_PART_B_DCHB_SONIPAT.pdf
22. International Institute for Population Sciences (IIPS), 2014. District Level Household and Facility Survey (DLHS-4), 2012-13: India. Haryana; District fact sheet, Sonepat: Mumbai: IIPS. Available at: https://nrhm-mis.nic.in/DLHS4/State%20and%20District%20Factsheets/.../Sonipat.pdf
23. State Fact Sheet-Haryana; National Family Health Survey 2015-2016. Available at: http://rchiips.org/NFHS/pdf/NFHS4/HR_FactSheet.pdf
24. Sample registration system (SRS) Bulletin; Registrar General of India (2016). Available at: http://www.censusindia.gov.in/vital_statistics/SRS_Bulletin_2015.pdf
25. PHFI, AIIMS, and SC- State of India's Newborns (SOIN) 2014- a report. (Eds) Zodpey S and Paul VK. Public Health Foundation of India, All India Institute of Medical Sciences and Save the Children. New Delhi, India. Available at: https://www.newbornwhocc.org/SOIN_PRINTED%2014-9-2014.pdf
26. Mony PK, Jayanna K, Bhat S, Rao SV, Crockett M, Avery L, et al. Availability of emergency neonatal care in eight districts of Karnataka state, southern India: a cross-sectional study. *BMC Health Serv Res* 2015; 15(1): 461
27. Annual Health Survey fact sheet; Uttar Pradesh (2012-13). Vital Statistics Division. Office of the Registrar General and Census Commissioner, Ministry of Home Affairs, Government of India. Available at: http://www.censusindia.gov.in/vital_statistics/AHSBulletins/AHS_Factsheets_2012-13/FACTSHEET-UTTAR_PRADESH.pdf
28. Michie, S., M.M. van Stralen, and R. West. The behaviour change wheel: a new method for characterising and designing behaviour change interventions. Implement Sci. 2011. 6: 42

For peer review only - http://bmjopen.bmj.com/site/about/guidelines.xhtml

29. Seidman G, Unnikrishnan S, Kenny E, Myslinski S, Cairns-Smith S, Mulligan B, et al. Barriers and enablers of Kangaroo Mother Care practice: a systematic review. PLOS ONE. 2015, 10(5):e0125643.
30. Chan GJ, Labar AS, Wall S, Atun R. Kangaroo mother care: a systematic review of barriers and enablers. Bulletin of the World Health Organization. 2016, 94(2):130-141J.
31. Smith ER, Bergelson I, Constantian S, Valsangkar B, Chan GJ. Barriers and enablers of health system adoption of kangaroo mother care: a systematic review of caregiver perspectives. BMC Pediatr. 2017; 17: 35.

For peer review only

For peer review only - http://bmjopen.bmj.com/site/about/guidelines.xhtml

	**Pre-KMC Facility**	**KMC Facility**	**Post-KMC Facility**
Health workers	Community health workers	Health facility staff: midwives, nurses, medical officer in charge, obstetricians, paediatricians	Community health workers
Activities	- Pregnancy surveillance<br>- Antenatal care visits<br>- Encouragement of facility births<br>- Birth notification<br>- Weigh all newborns<br>- Support cultural norms endorsing KMC<br>- Referral of newborns <2000 g to a KMC facility	- Weigh all newborns<br>- Accept referrals<br>- **Initiate KMC** for all newborns <2000 g<br>- Maintain KMC<br>- Breastfeeding support<br>- Quality Improvement to ensure effective KMC	- Scheduled home visits within 24 hours of discharge and on days of life 7 and 28<br>- **KMC continuation** at home<br>- Breastfeeding support<br>- Identification and referral of sick newborns or newborns unable to continue KMC
Outputs	- Identify KMC eligible newborns (<2000 g)<br>- Get all newborns <2000 g to KMC facility	- Effective KMC initiated and maintained for all newborns <2000 g	- Effective KMC continued at home

Health system strengthening

- Linkage of KMC facilities and communities
- Referral System to identify KMC eligible (<2000 g) and/or sick newborns, and facilitate their transfer from the community to KMC facility

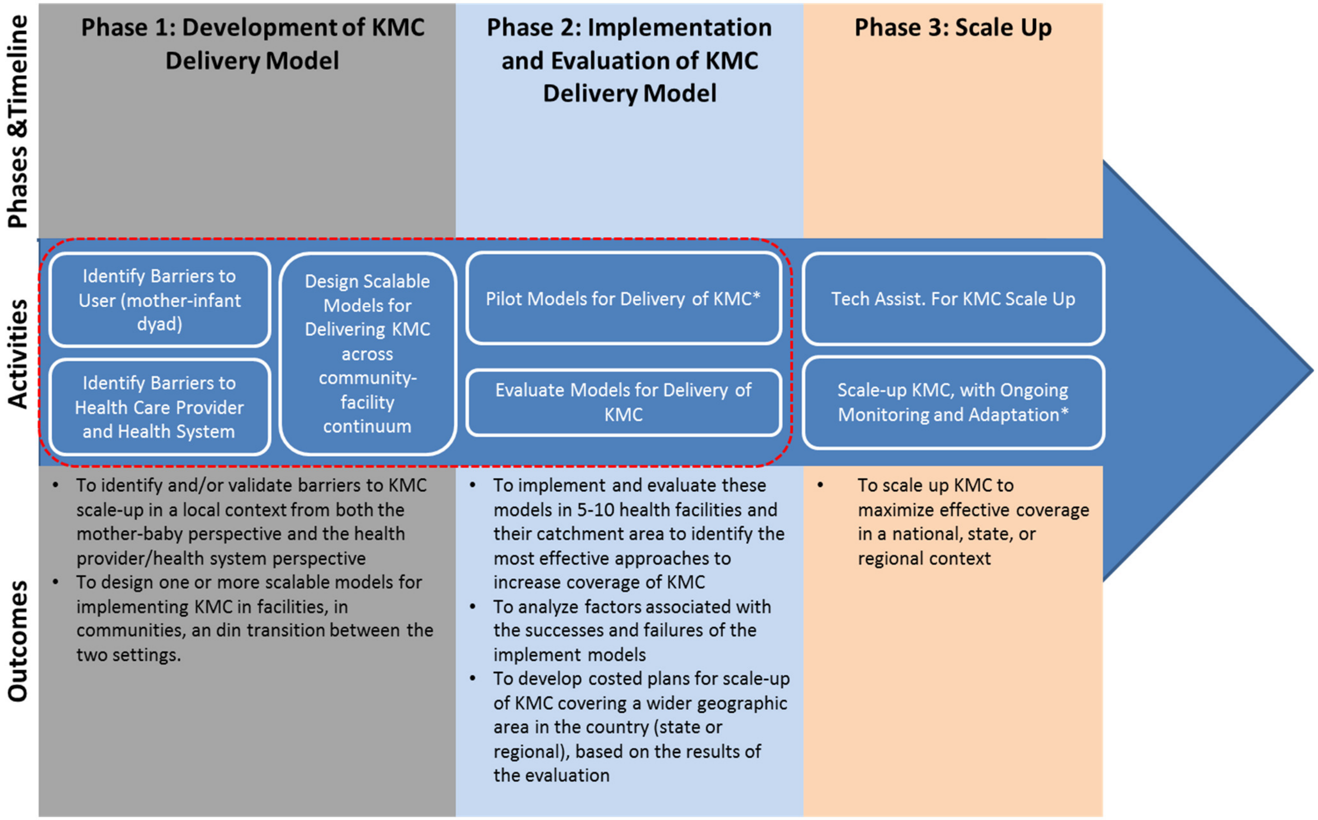

- These activities to be implemented by government with long-term accountability for KMC provision
- Scope of protocol - - - - - - - - - - - - - - - -

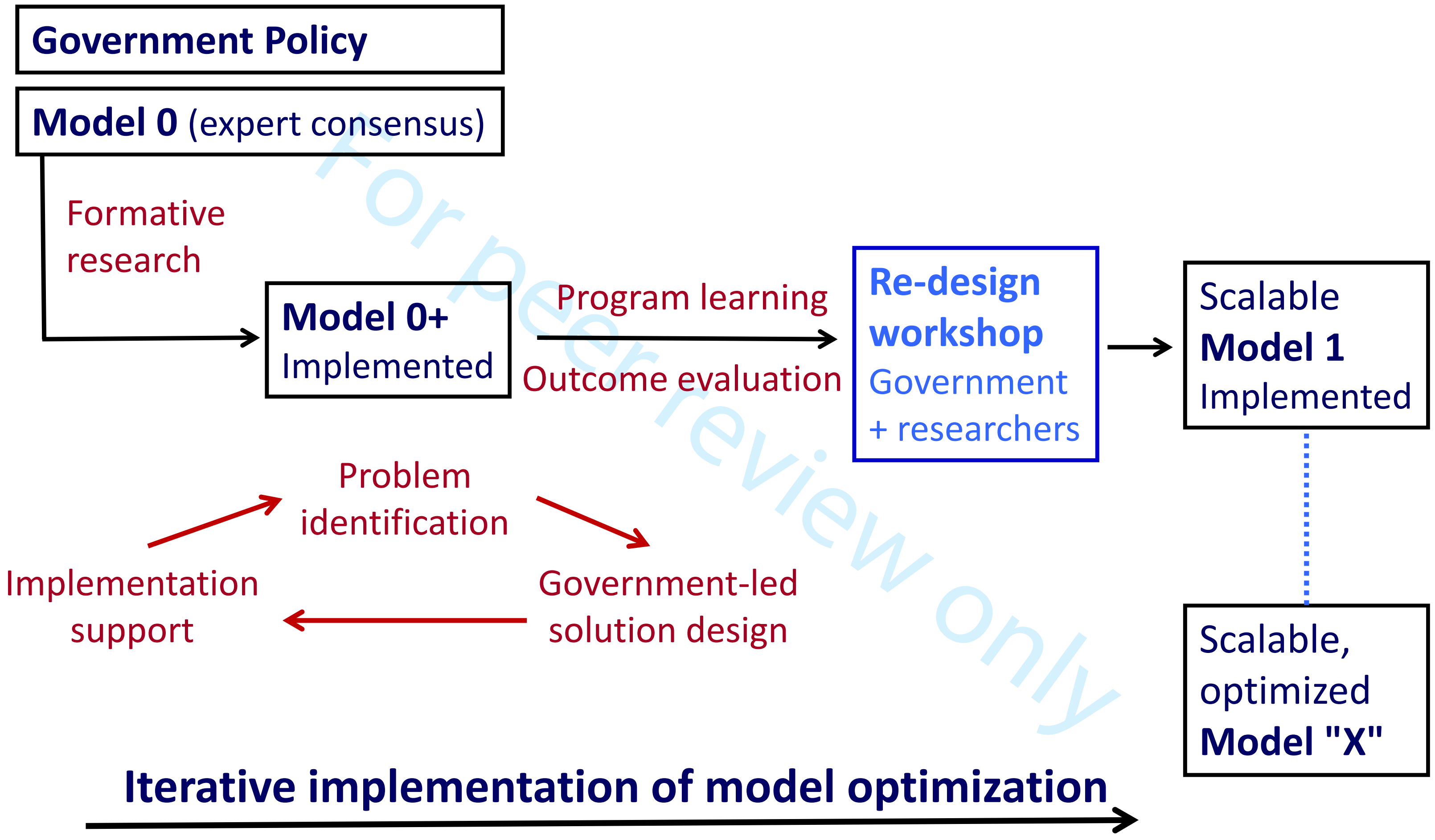

For peer review only

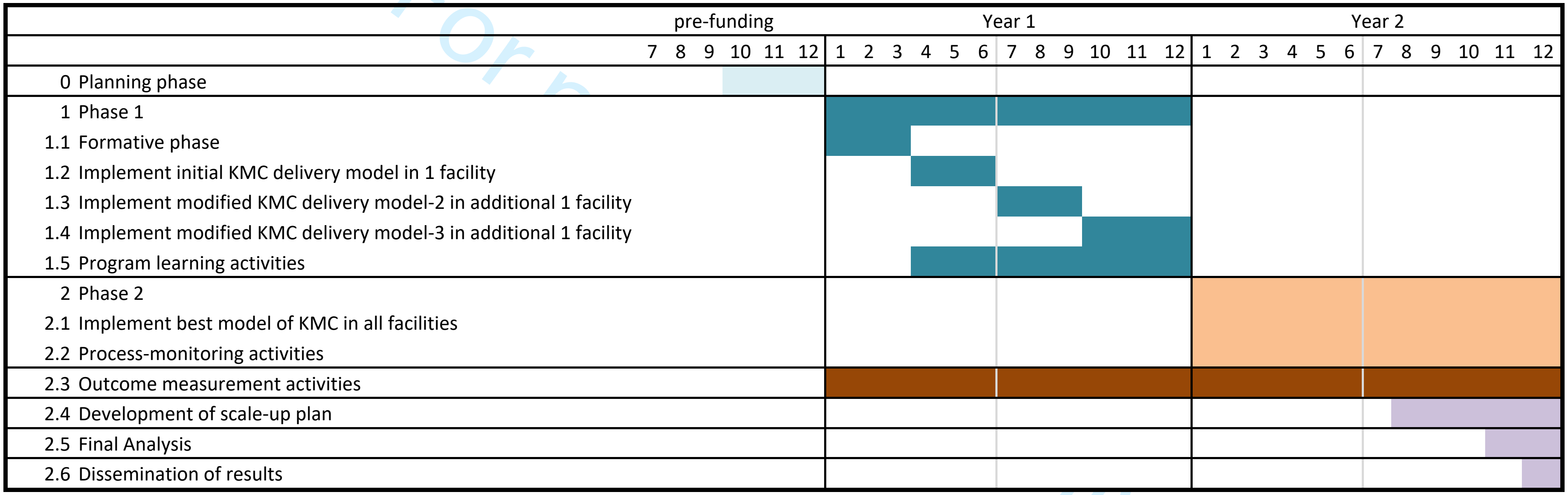
pre-funding
Year 1
Year 2
7 8 9 10 11 12
1 2 3 4 5 6 7 8 9 10 11 12
1 2 3 4 5 6 7 8 9 10 11 12
0 Planning phase
1 Phase 1
1.1 Formative phase
1.2 Implement initial KMC delivery model in 1 facility
1.3 Implement modified KMC delivery model-2 in additional 1 facility
1.4 Implement modified KMC delivery model-3 in additional 1 facility
1.5 Program learning activities
2 Phase 2
2.1 Implement best model of KMC in all facilities
2.2 Process-monitoring activities
2.3 Outcome measurement activities
2.4 Development of scale-up plan
2.5 Final Analysis
2.6 Dissemination of results

BMJ Open

Kangaroo Mother Care Implementation Research to Develop Models for Accelerating Scale-up in India and Ethiopia: Study Protocol for an Adequacy Evaluation

Journal:	*BMJ Open*
Manuscript ID	bmjopen-2018-025879.R2
Article Type:	Protocol
Date Submitted by the Author:	10-Sep-2019
Complete List of Authors:	Medhanyie , Araya; Mekelle University College of Health Sciences, School of Public Health<br>Alemu, Hilbret; John Snow Inc, Urban Health<br>Asefa, Anteneh; Hawassa University College of Medicine and Health Sciences, School of Public Health<br>Beyene , Selemawit; Mekelle University College of Health Sciences, School of Public Health<br>Gebregizabher , Fisseha; Tigray Regional Health Bureau<br>Aziz, Khalid; University of Alberta, Department of Neonatology<br>Bhandari, Nita; Society for applied studies, Centre for Health Research and Development<br>Beyene, Habtamu; SNNPR Regional Health Bureau<br>Brune, Thomas; Karloniska Institute, Department of Neonatology<br>Chan, Grace; Harvard Medical School; Boston Children's Hospital , Division of Medicine Critical Care<br>Cranmer, John; Emory University, Nell Hodgson Woodruff School of Nursing<br>Darmstadt, G; Stanford University School of Medicine, Department of Pediatrics<br>Duguma, Dereje; Oromia Regional Health Bureau<br>Fikre, Addisalem; Addis Ababa University School of Public Health<br>Andualem, Bizuayehu; Amhara National Regional Health Bureau<br>Gobezayehu, Abebe; Emory University Ethiopia<br>Mariam, Damen; Addis Ababa University School of Public Health<br>Abay, Tedros; Mekelle University College of Health Sciences, Department of Pediatrics and Child Health<br>Mohan, H L; Karnataka Health Promotion Trust, Community Mobilization<br>Jadaun, Arun; Society for Applied Studies, Centre for Health Research and Development<br>Jayanna, K; Karnataka Health Promotion Trust, Bangalore, Karnataka, India, Quality Improvement<br>Kajal, FNU; Indian Administrative Service, National Health Mission<br>Kar, Arin; Karnataka Health Promotion Trust, Department of Health & Family Welfare<br>Krishna, Raghav ; Community Empowerment Lab, Global Health<br>Kumar, Aarti; Foundation for Community Empowerment, Global Health<br>Kumar, Vishwajeet ; St. John's Medical College & Research Institute, Community Health and Epidemiology<br>Madhur, Tarun; Society for Applied Studies, Centre for Health Research

	and Development<br>Belew, Mulusew; Emory University, Amhara Regional Office<br>M, Rajini; Government of Karnataka, Department of Health & Family Welfare<br>Martines, Jose; Universitetet i Bergen Senter for internasjonal helse, Centre for Intervention Science in Maternal and Child Health<br>Mazumder, Sarmila; Society for Applied Studies, Centre for Health Research and Development<br>Amin, Hajira; Emory University , M&E<br>Mony, Prem; St. John's Research Institute, St. John's National Academy of Health Sciences, Division of Epidemiology & Population Health<br>Muleta, Mekonnen; Private Consultant<br>Pileggi-Castro, Cynthia; Centro de Referência da Saúde da Mulher de Ribeirão Preto, Maternidade<br>Pn Rao, Suman; St John's Medical College Hospital, Department of Neonatology<br>Estifanos, Abiy; Addis Ababa University School of Public Health<br>Sibley, Lynn M.; Emory University School of Public Health, Global Health<br>Singhal, Nalini; University of Calgary Cumming School of Medicine, Department of Neonatology<br>Tadele, Henok; Hawassa University<br>Tariku, Abraham; Federal Ministry of Health<br>Lemango , Ephrem; Federal Ministry of Health , Maternal and Child Health Directorate<br>Tadesse, Birkneh; Hawassa University College of Medicine and Health Sciences, Department of Pediatrics and Child Health<br>Upadhyay, Ravi; Centre for Health Research and Development,<br>Worku, Bogale; Pediatrics Society; Addis Ababa University<br>Hadush, Marta; Mekelle University College of Health Sciences, Department of Pediatrics and Child Health<br>Bahl, Rajiv; World Health Organization

**Primary Subject Heading**:	Health services research
Secondary Subject Heading:	Paediatrics, Global health
Keywords:	kangaroo-mother care method, low birth weight, newborn, research protocol

SCHOLARONE™
Manuscripts

TITLE PAGE

KANGAROO MOTHER CARE IMPLEMENTATION RESEARCH TO DEVELOP MODELS FOR ACCELERATING SCALE-UP IN INDIA AND ETHIOPIA: STUDY PROTOCOL FOR AN ADEQUACY EVALUATION

KMC Scale-Up Study Group*

Araya Abrha Medhanyie [a], Hibret Alemu[b], Anteneh Asefa[c], Selemawit Asfaw Beyene[a], Fisseha Ashebir Gebregizabher[d], Khalid Aziz[e], Nita Bhandari[f], Habtamu Beyene[g], Thomas Brune[h], Grace J Chan[i], John N Cranmer[j], Gary Lee Darmstadt[k], Dereje Duguma[l], Addisalem Fikre[m], Bizuayehu Gashaw Andualem[n], Abebe Gebremariam Gobezayehu[o], Damen Haile Mariam[m], Tedros Hailu Abay[a], Mohan HL[p], Arun Jadaun[f], Krishnamurthy Jayanna[p], Kajal (no last name)[r], Arin Kar[p], Raghav Krishna [q], Aarti Kumar [q], Vishwajeet Kumar [q], Tarun Kumar Madhur[f], Mulusew Lijalem Belew[o], Rajini M[s], Jose Carlos Martines[t], Sarmila Mazumder[f], Hajira Mohammed Amin[o], Prem K. Mony[u], Mekonnen Muleta[v], Cynthia Pileggi-Castro [w], Suman Rao[x], Abiy Seifu Estifanos[m], Lynn M Sibley[j], Nalini Singhal[y], Henok Tadele[c], Abraham Tariku[z], Ephrem Tekle Lemango[z], Birkneh Tilahun Tadesse[c], Ravi Prakash Upadhyay[f], Bogale Worku[m,aa], Marta Yemane Hadush[a], Rajiv Bahl[ab]

[a]Mekelle University, Mekelle, Ethiopia

[b]JSI Urban Health, Addis Ababa, Ethiopia

[c]Hawassa University, Hawassa, Ethiopia

[d]Tigray Regional Health Bureau, Mekelle, Ethiopia

[e]University of Alberta, Edmonton, Canada

[f]Centre for Health Research and Development, Society for Applied Studies, New Delhi, Delhi, India

[g]Southern Nations, Nationalities, and Peoples' Regional Health Bureau, Hawassa, Ethiopia

[h]Karloniska Institute, Stockholm, Sweden

[i]Harvard Medical School and Harvard T.H. Chan School of Public Health.

[j]Emory University, Atlanta, GA, USA

[k]Stanford University School of Medicine, Stanford, CA, USA

[l]Oromia Regional Health Bureau, Addis Ababa, Ethiopia

[m]Addis Ababa University, Addis Ababa, Oromia, Ethiopia

[n]Amhara Regional Health Bureau, Bahir Dar, Ethiopia

[o]Emory Ethiopia, Addis Ababa, Ethiopia

[p]Karnataka Health Promotion Trust, Bangalore, India

For peer review only - http://bmjopen.bmj.com/site/about/guidelines.xhtml

Kangaroo Mother Care implementation research protocol

[q]Community Empowerment Lab, Global Health, Lucknow, India
[r]Indian Administrative Service, National Health Mission, Lucknow, India
[s]Department of Health & Family Welfare, Government of Karnataka, Bangalore, India
[t]Universitetet i Bergen, Centre for Intervention Science in Maternal and Child Health, Bergen, Norway
[u]St John's Research Institute, Bangalore, India
[v]Private Consultant, Addis Ababa, Ethiopia
[w]Centro de Referência da Saúde da Mulher de Ribeirão Preto, Maternidade, Ribeirão Preto (Sao Paolo), Brazil
[x]St. John's Medical College Hospital, Bangalore, India
[y]University of Calgary, Calgary, Canada
[z]Federal Ministry of Health, Addis Ababa, Ethiopia
[aa]Ethiopian Pediatrics Society, Addis Ababa, Ethiopia
[ab]World Health Organization

Corresponding author: Rajiv Bahl

Email: bahlr@who.int

Keywords: kangaroo-mother care method; infant, low birth weight; infant, newborn; research

Word Count: ~4600

Financial disclosure statement: The authors have no financial relationship relevant to this article to disclose

Funding / Support: The World Health Organization, the sponsor, will fund the study with financial support from the Bill and Melinda Gates Foundation. WHO facilitated the preparation of the protocol, will monitor implementation and analysis of data. It will also facilitate the writing of the report and participate in the decision to submit the report for publication. The study teams will have ultimate authority over any of these activities.

Potential conflicts of interest: The authors have no conflicts of interest relevant to this article to disclose.

For peer review only - http://bmjopen.bmj.com/site/about/guidelines.xhtml

Clinical Trial registry name and registration number:

Community Empowerment Laboratory, Uttar Pradesh, India - ISRCTN12286667

St John's National Academy of Health Sciences, Bangalore, India and Karnataka Health Promotion Trust, Bangalore, India - CTRI/2017/07/008988.

Society for Applied Studies, Delhi - NCT03098069

Oromia, Ethiopia - NCT03419416.

Amhara, SNNPR and Tigray, Ethiopia - NCT03506698.

List of abbreviations

CHW- community health worker

HEW- Health extension worker

KMC- Kangaroo mother care

LBW- Low birth weight

L10K- Last ten kilometers project

NICU- Neonatal intensive care unit

QA- Quality assurance

QI- Quality improvement

SNCU- Special neonatal care unit

SNNPR- Southern Nations Nationalities and People's Region

UP- Uttar Pradesh

WHO- World Health Organization

Short running title: Kangaroo Mother Care implementation research protocol

For peer review only

For peer review only - http://bmjopen.bmj.com/site/about/guidelines.xhtml

ABSTRACT

Introduction: Kangaroo Mother Care (KMC) is the practice of early, continuous and prolonged skin-to-skin contact between the mother and the baby with exclusive breastfeeding. Despite clear evidence of impact in improving survival and health outcomes among low birth weight infants, KMC coverage has remained low and implementation has been limited. Consequently, only a small fraction of newborns that could benefit from KMC receive it.

Methods and analysis: This implementation research project aims to develop and evaluate district-level models for scaling up KMC in India and Ethiopia that can achieve high population coverage. The project includes formative research to identify barriers and contextual factors that affect implementation and utilization of KMC and design scalable models to deliver KMC across the facility-community continuum. This will be followed by implementation and evaluation of these models in routine care settings, in an iterative fashion, with the aim of reaching a successful model for wider district, state and national-level scale-up. Implementation actions would happen at three levels: “pre-KMC facility” - to maximize the number of newborns getting to a facility that provides KMC; “KMC facility” - for initiation and maintenance of KMC; and “post-KMC facility” - for continuation of KMC at home. Stable infants with birth weight <2000 g and born in the catchment population of the study KMC facilities would form the eligible population. The primary outcome will be coverage of KMC in the preceding 24 hours and will be measured at discharge from the KMC facility and seven days after hospital discharge.

Ethics and dissemination: Ethics approval was obtained in all the project sites, and centrally by the Research Ethics Review Committee at the World Health Organization. Results of the project will be submitted to a peer-reviewed journal for publication, in addition to national and global level dissemination.

Strengths and limitations of the study

- This will be the first implementation research project to develop and evaluate models for achieving high population coverage with KMC.
- The project is a partnership between research groups and local governments, implemented at large scale, covering populations in diverse regions in two countries, Ethiopia and India, of high prevalence of low birth weight.

For peer review only - http://bmjopen.bmj.com/site/about/guidelines.xhtml

- This study has a significant limitation, the lack of a concomitant comparison group. The models' success in increasing KMC coverage will be assessed through the comparison with data at the beginning of the project that indicated that KMC is a rare practice in these settings, covering under 5% of low birth weights, and that competing intervention promoting KMC are not currently implemented in study sites.

For peer review only

For peer review only - http://bmjopen.bmj.com/site/about/guidelines.xhtml

INTRODUCTION

Globally, each year 15 million neonates are born preterm and consequently at a high risk of mortality.[1,2] South Asia and sub-Saharan Africa account for almost two-thirds of the world's preterm babies.[3] Kangaroo Mother Care (KMC) is a low-cost intervention involving early, continuous and prolonged skin-to-skin contact between mother and baby and exclusive breastfeeding.[4] KMC has been demonstrated to promote physiologic stability, a thermally-supportive environment, reduce risk of serious infections, and reduce the mortality among hospitalized, stable preterm and low birth weight (LBW) infants.[5] Despite the benefits, coverage of KMC has remained low and implementation has largely been limited to specialized hospitals.[6-8] As a result, only a small fraction of newborns that could benefit from KMC receive it. A set of seven studies has been planned in Ethiopia and India to develop and test models of scaling-up KMC effectively.

AIMS OF THE IMPLEMENTATION RESEARCH

Aim: To develop a delivery model that will result in high coverage (80%+) and quality of KMC for the target population.

Additional aims:

A: To design and identify the most promising delivery model through formative research combined with an iterative quality improvement (QI) approach.

B: To implement the most promising model in the study facilities and evaluate quality and coverage of KMC in the population they serve.

METHODS/ DESIGN

We are undertaking an international, multi-site, implementation study using a mixed-methods design that integrates formative and evaluative components into iterative improvement cycles. Our intervention promotes KMC for babies born <2000 g across the community-facility continuum.

Population

The population will be newborns with birth weight under 2000 g born in the geographic area targeted by the intervention. It includes all babies born in health facilities and in the community. For those

For peer review only - http://bmjopen.bmj.com/site/about/guidelines.xhtml

newborns who are severely sick according to pre-defined criteria (not tolerating oral feeds, severe respiratory distress including, respiratory rate less than 20 breaths per minute, grunting, central cyanosis, severe chest indrawing, convulsions, unconsciousness, and severe hypothermia of less than 32 degrees Celsius), KMC will be delayed until they stabilized.

Intervention

The intervention will be the provision of KMC, defined as skin-to-skin contact with the mother or a replacing caregiver, for as long as possible during day and night, with a minimum of at least eight hours over a 24-hour period, along with exclusive breastfeeding or breast milk feeding via tube, spoon or paladai. Based on the available evidence of effectiveness, KMC will only be initiated in health facilities.[5]

Comparison

The performance of the interventions will be assessed against a predefined success criterion of 80% or higher coverage of effective KMC at population level.

Outcomes

The assumption that current coverage with KMC initiated at a health facility is low or negligible in the study population will be confirmed by a baseline assessment of facilities.

The primary outcome will be effective coverage of KMC, defined as at least eight hours of skin-to-skin contact in combination with exclusive breastfeeding in the preceding 24 hours among infants with birth weight <2000 g. It will be measured:

- at discharge from the KMC facility (defined as one where staffing, infrastructure and mechanisms are present to initiate and maintain KMC)
- at 7 days after discharge, in the home
- at 28 days of age, in the home

Secondary outcomes include:

— Population-level duration of KMC, i.e. mean of how many days KMC was received by infants in the population with birth weight <2000 g during the neonatal period.

For peer review only - http://bmjopen.bmj.com/site/about/guidelines.xhtml

- Proportion of infants with birth weight <2000 g receiving any KMC (any duration in last 24 hours) at discharge, seven days post discharge and at 28 days of age
- Proportion of infants with birth weight <2000 g exclusively breastfed (based on 24-hour recall) at discharge, seven days post discharge and at 28 days of age
- Neonatal mortality rate (including early neonatal mortality rate) in infants <2000 g
- Cost of the KMC model (measured in some of the sites)

Sample Size

We assume that 3-5% of neonates born in Ethiopia and India will be <2000 g and that it will not be possible to evaluate 20% of these newborns because of early death or loss to follow up. A sample size of 310 newborns per site will allow us to estimate 80% coverage of KMC with site-specific absolute precision of ±5%. All the study sites will have sufficient numbers of births of eligible infants to enrol at least 310 during the evaluation phase. We anticipate the follow-up of a total of at least 2170 newborns <2000 g during the evaluation period.

Study Sites

The study will be conducted in a total of seven sites in Ethiopia and in India. Ethiopia and India are among the countries in Africa and Asia with the highest proportion of low birth weight babies. Selection of sites within each country considered the quality of the proposals and the desire to reflect regional diversity.

Ethiopia

In Ethiopia, newborn mortality remains high at 29 per 1000 live births.[9,10] The Ministry of Health has spearheaded a major campaign to increase facility births which appears effective.[11-15] Results from a recent survey (L10K) in 2015 conducted in 115 districts (woredas) in agrarian regions, indicate facility births in the past 12 months to be around 53%.[11]

Amhara

The study will be conducted in two woredas of West Gojam Zone, two woredas of South Gondar Zone, and Bahir Dar City Administration, Amhara Region. In West Gojam zone, the study includes one primary hospital. In Bahir Dar City Administration, the study includes a large referral hospital, one primary hospital, 19 health centres, 42 health posts and their respective catchment populations. In South Gondar Zone, the study includes one general hospital and one primary

For peer review only - http://bmjopen.bmj.com/site/about/guidelines.xhtml

hospital, 14 health centres and up to 59 health posts affiliated with these health centres, along with their respective catchment populations.

Oromia – Addis Ababa

The Oromia – Addis Ababa site will implement the study in Akaki Kality sub-city in Addis Ababa and East Shoa zone in Oromia region. Akaki Kality sub-city, one of the 10 sub-cities of Addis Ababa city administration shares border and culture with East Shoa zone of Oromia region. The Akaki Kality sub-city has an estimated population of 180,000 living in 11 woredas in the sub-city.[14] Oromia Regional state has an estimated population of 27 million [14]. The study will be implemented in two woredas: Adami Tulu in the East Shewa Zone and Arsi Robe in the Arsi Zone.

SNNPR

SNNPR has a facility birth rate of 25.2%.[10] The sites proposed for study are Hawassa city administration, Shebedino woreda and Dale woreda, situated in Sidama zone. Hawassa is the capital of SNNPR and its administration consists of eight sub-cities with urban and rural kebeles. The two public hospitals in Hawassa city—namely Hawassa University Comprehensive Specialized Hospital and Adare General Hospital—are included in the study. Leku is the capital of Shebedino woreda and consists of urban and rural kebeles. Leku Primary Hospital is the site for Shebedino woreda. Yirgalem is the capital of Dale woreda. Yirgalem General Hospital is the site for Dale woreda.

Tigray

According to the EDHS 2016, in the Tigray region, 69% of the women who gave birth two years preceding the survey delivered in facilities. The study will be implemented in two zones, namely south-east zone and Mekelle special zone. In the south-east zone, two districts (Enderta and Degu-Tembien woredas) will be included in the study. In the selected districts and Mekelle Zone, there are one comprehensive referral hospital, two general hospitals, one primary hospital, 22 health centres and five private health facilities. All these health facilities will be included in the study

For peer review only - http://bmjopen.bmj.com/site/about/guidelines.xhtml

India

India accounts for one quarter of newborn deaths in the world. Neonatal deaths represent about 50% of under-five deaths and 70% of its infant deaths.[16] About 27% of all babies are low birth weight (<2500g).[17-20]

Haryana

The study will be implemented in one of Haryana's 22 districts (Sonepat). The low birth weight rate is 18% in Sonepat.[21,22] Around 5% of all infants weigh less than 2000 g at birth (*information from government records*). The institutional delivery rate is 80.5% and 90% of births are conducted by skilled birth attendants.[23] Infant mortality in Haryana is 36/1000, similar to the Indian average.[24] The neonatal mortality rate (NMR) is 28/1000, a major proportion of infant mortality rate.[25]

Karnataka

Infant mortality rate is 28/1000 and is NMR is 22/1000 live births.[24,25] The research project will be conducted in the Koppal district, located 400 km away from Bangalore. This is an under-served, in the northern region of the state identified as a high priority district by the government.[26] The district has a low birth weight prevalence of about 25% and a NMR of 42/1000. About 80% of births are institutional deliveries.

Uttar Pradesh (UP)

UP is the most populous state in India and accounts for a quarter of India's burden of neonatal deaths.[25] UP's NMR of 37/1000 is amongst the highest in the country.[25] The prevalence of low birth weight in UP is 25%.[27] The study will be implemented in the Raebareli district with an institutional delivery rate of 93%. The study will cover the district hospital and ten community health centres, along with their catchment blocks.

Ethical Considerations

Since the intervention will be provided by the government as standard of care for LBW, there will be no request for consent from individuals to receive the intervention. Individual written informed consent will be requested from mothers, caregivers, and health workers for the collection of information. For those unable to read, the informed consent form will be read out by the research team member before asking consent.

Patient and Public Involvement

For peer review only - http://bmjopen.bmj.com/site/about/guidelines.xhtml

The question addressed by this research project was identified as a priority by the governments of Ethiopia and India. They participated in planning the study through the ministries of health. Patients and communities will play a major role in designing the models developed and tested, as described below under Phase 1 – Formative research. Results will be disseminated to the communities using channels available to the health system, as well as to the state and national health authorities, and through publication in peer-reviewed journals.

Study approvals

Study approvals were obtained at the local, national and international levels. Ethics approvals were taken from the ethics committees of the institutions engaged in the project and centrally from the World Health Organization (WHO) Ethics Committee.

Study implementation strategy

Core Framework for KMC Implementation

KMC promotion will be implemented at three levels (Figure 1):

INSERT FIGURE 1 HERE

"Pre-KMC facility" - the aim at this level is to maximize the proportion of newborns under 2000 g to be born in/brought to KMC implementing health facilities.

"KMC facility" – the aim at this level is early initiation of KMC and adherence to long duration (>8 hours) skin-to-skin care with exclusive breastfeeding within the facility.

"Post-KMC facility" – the aim at this level is continuation of effective KMC at home through the neonatal period or until the baby no longer accepts it.

Study Teams

The KMC intervention will be delivered by health workers who are part of the health system. The research team's implementation role will be only supportive. This research team will be comprised of three small teams:

(i) The "program learning team", to conduct formative research and process-monitoring, help formulate the intervention and process-evaluation of implementation.

For peer review only - http://bmjopen.bmj.com/site/about/guidelines.xhtml

(ii) The "implementation support team" to guide and support the health system so that the environment supports KMC and health workers implementing the intervention.

(iii) The "outcome evaluation team" to measure the study outcomes in the population acting independently from the other two teams (Table 1).

For peer review only

For peer review only - http://bmjopen.bmj.com/site/about/guidelines.xhtml

Table 1. Description of Study Implementation Teams

	Research			Government Implementation
	Program learning team	Implementation support team	Outcome evaluation team	Health system team
Phase 1	Conduct formative research to develop the initial KMC delivery model.<br><br>Conduct program learning to understand how well the KMC delivery model is working and to redesign the model based on lessons learned.	Support government implementation of KMC.	In the catchment area of the facilities involved in Phase 1, collect quantitative data on:<br>• Births in facilities and community<br>• Births <2000 g in facilities and community<br>• Number of newborns receiving KMC at time of discharge from health facility, seven days post discharge and at 28 days of age<br>• Duration of skin-to-skin care	Deliver and monitor the KMC intervention.

For peer review only - http://bmjopen.bmj.com/site/about/guidelines.xhtml

Phase 2	Monitor process indicators to learn how implementation of KMC delivery models happens in all study facilities and identify ways to refine the model based on context-specific findings.	Assume an advisory role to support government implementation of KMC.	In the entire study population, collect quantitative data as above.	Deliver and monitor the KMC intervention.

For peer review only

For peer review only - http://bmjopen.bmj.com/site/about/guidelines.xhtml

Phases of implementation

The implementation will happen in two phases (Figure 2).

INSERT FIGURE 2 HERE

Phase 1: To Develop a Delivery Model for KMC

KMC Delivery Model Cycles of Improvement

The initial delivery model will be developed based on the formative research and synthesis of literature. Program learning from the delivery of this model and outcome measurements will immediately feed into a stage of model review and redesign/refinement, aimed at improving the coverage. We expect that at least three cycles of model development-implementation-evaluation-refinement will be needed until reaching a scalable high coverage model. Each cycle may take three to four months. The first cycle may involve one facility but the second and third will be larger, including up to one-third of all study facilities.

At the end of each cycle, findings will be shared with an advisory group of stakeholders and their input will be sought to improve the model. New iterations of the model will be developed and further refined and reviewed with advisors. Figure 3 presents this iterative implementation-optimization of the model.

INSERT FIGURE 3 HERE

Formative research

The study's first step will be the program learning research team undertaking formative research to identify and understand barriers and enablers to KMC in the specific context. We will follow the COM-B model to guide understanding of behaviour change in the implementation context, and the development of behavioural targets as a basis for designing interventions with users[28]. It proposes that people need capability (C: psychological or physical ability to enact a behaviour), opportunity (O: physical and social environment that enable a behaviour) and motivation (M: reflective and automatic mechanisms that activate or inhibit a behaviour) to perform a behaviour (B). Domains of the formative research are presented in Table 2. Based on the knowledge gathered, we will develop an initial model of KMC implementation at the study site.

For peer review only - http://bmjopen.bmj.com/site/about/guidelines.xhtml

Implementation of KMC test models

The initial model will be implemented in collaboration with facility and community workers with guidance from the implementation support team. Selection of facilities to implement KMC test models will be based on factors such as number of births, type of health workers, services delivered (e.g. presence of newborn intensive care units or special care nurseries) so that they capture a major proportion of deliveries in the study district. Implementation will occur at three levels: pre-KMC facility, KMC facility, and post-KMC facility (Figure 1).

For peer review only

For peer review only - http://bmjopen.bmj.com/site/about/guidelines.xhtml

Table 2. Domains of Enquiry for Formative Research

Domains of enquiry	Activities
**KMC Intervention** – to inform the design of the technical package of KMC	• Review KMC scientific literature; existing government guidelines on KMC; experience on implementation of KMC<br>• Develop guidelines and protocols for health staff in facility and community health care workers<br>• Develop communication materials for communities, pregnant women and their families
**Users and implementers** – to understand the barriers and enablers to KMC adoption and adherence	**Mothers/Families**<br>• Explore perceptions of mothers and family members around preterm babies, low birth weight, care-seeking, institutional deliveries, private facilities, perception of quality; understand and explore ideas to generate demand for KMC<br>**Health Workers**<br>• Assess understanding and experience of KMC; understanding of birth weight, prematurity<br>• Assess knowledge, skill, time and motivation for newborn care
**Health systems** – to improve the delivery of KMC at the facility and community level	**Health facilities**<br>• Understand current KMC practices in health facilities; map behaviours that could impact adoption of KMC within these facilities<br>• Design a KMC facility readiness checklist based on local feasibility and provisions<br>• Assess physical infrastructure and resources available for KMC<br>**Link between community and facility**<br>• Assess current engagement between CHWs and health facilities and explore efficient process of engagement<br>• Assess referral system<br>**Assess data needs, measurement systems and analytics support and its compatibility and adaptability for KMC**<br>• Assess existing management information system; assess registries that exist at each level of the health care system<br>• Design and test electronic data management systems to guide and accelerate adoption and improvement of KMC<br>**Leadership and policy makers**

For peer review only - http://bmjopen.bmj.com/site/about/guidelines.xhtml

	• Engage leadership and decision makers for their support of KMC<br>• Assess need of additional workers and current human resource capacity gap<br>**Other stakeholders**<br>• Map and engage all professional, organizational, health system, administrative and social networks that may impede or support KMC adoption and spread in the study area
**Context** – to develop adaptable delivery models	• Understand perceptions of care of small babies and logistical challenges to care of high risk babies within the setting<br>• Explore motivations for facility versus home deliveries<br>• Map relationships between doctors and nurses, CHWs, and mothers/families

For peer review only - http://bmjopen.bmj.com/site/about/guidelines.xhtml

Pre-KMC facility

To effectively implement KMC, eligible newborns need to be identified and cared for in facilities where KMC is initiated. All births should be identified, and babies weighed. Eligible babies, if born outside KMC facilities, will be referred to these facilities through strengthened government transport and linkage mechanisms. The pre-facility level includes efforts to: increase facility deliveries, expand early assessment of those delivered at home, so that eligible newborns are referred to a KMC facility, and create a social norm around the practice of KMC.

KMC facility

Facility level activities will aim to strengthen knowledge and skills of health providers and implement KMC protocols so that they provide high-quality care. All stable newborns would be placed in skin-to-skin contact and initiated breastfeeding within the first hour of life. In-born and referred newborns would be screened for KMC eligibility (< 2000 g and stable). If meeting eligibility criteria, KMC would be initiated. If the newborn is sick, he/she will be stabilized first and then reassessed for KMC eligibility and initiation.

During the newborn's facility stay, nurses will do a KMC assessment following a checklist which examines skin-to-skin contact (number of hours) and breastfeeding, counselling on KMC, and problem-solving if barriers exist. Nurses will document the adoption of KMC. At time of discharge, mothers and family members will be counselled so that KMC is continued at home. Facility staff will notify community health workers (CHWs) about discharges and discuss how to support continued KMC at home.

Post-KMC facility

After discharge, CHWs shall visit the home within 24 hours following the post-facility KMC protocol. CHWs will continue to visit the newborn following the standard national postnatal care visit schedule. At each visit, CHWs will perform a KMC assessment using the follow-up checklist including skin-to-skin duration and breastfeeding, counselling and problem solving. CHWs will identify and refer sick newborns to the facility and assist the referral.

Health systems strengthening

Linkages between facility and community will be strengthened so that KMC is continued at home. This will be facilitated through establishment of communication systems between facilities and CHWs.

For peer review only - http://bmjopen.bmj.com/site/about/guidelines.xhtml

Outcome Measurement

The study outcome evaluation team will conduct an independent evaluation of the effectiveness of the model in achieving 80% or higher coverage with KMC among the target population. Data will be collected at health facilities and the community.

Phase 2: Implement and Evaluate the chosen KMC Delivery Model

The most promising KMC delivery model will be chosen from Phase 1 based on program learning findings and coverage. During Phase 2, the chosen model will be implemented in all selected facilities and data will be collected in the study area to measure the coverage with effective KMC.

Implementation of the KMC Delivery Model

The model will include KMC guidelines, protocols, communication and training materials, and tools for successful implementation. The delivery model will be implemented by the health system in all study facilities and their catchment areas. The implementation support team will continue to be active but now playing only an advisory role to the government implementation teams. There will be ongoing mentorship and quality improvement feedback cycles within each facility.

Implementation activities will include guideline dissemination, health care provider and CHW training, identification of low birth weights, community awareness and demand generation, establishment of referral and supervision systems, monitoring, and continuous feedback loops. Key activities are further described below.

Community awareness and demand generation: Contacts between families the health services will be utilized to promote awareness of KMC and of KMC units. Pregnant women and mothers of under-fives, for example, come in contact with health services during antenatal care, immunization sessions and routine home visits by CHWs. Additional activities, such as village health promotion days, will be used. Messages developed will be communicated through posters, pamphlets, banners and wall paintings and disseminated at strategic locations in the community and at facilities with maternity wards, antenatal clinics, postnatal clinics, paediatrics wards in addition to the KMC wards. Audiovisual media such as local network for television channels or local radio may also be used.

Consistent messages from multiple channels including interpersonal communication with health providers in public and private sectors and CHWs, will reinforce the information. Experience indicates that the endorsement from doctors seems critical to adoption of KMC. Orientation sessions will be conducted with all stakeholders and community representatives likely to influence community behaviour and norms.

For peer review only - http://bmjopen.bmj.com/site/about/guidelines.xhtml

Identification of resources and staffing within the health system: KMC is a new intervention for most health facilities in Ethiopia and India. Before initiating field work, discussions with national and local government officials will be held to promote buy-in. During these meetings, a landscaping overview of staffing, equipment and supplies at study health facilities will be completed. Together with government partners, research teams will meet with the leadership at study health facilities to introduce the KMC initiative, discuss their concerns and engage their involvement. We will jointly identify staff to participate in KMC implementation at each level of the health system.

Development of intervention materials: With Ministries of Health and country-specific teams, we will develop a standardized set of KMC protocols, guidelines, training materials, job tools, and checklists to support implementation. Existing training manuals on newborn care in Ethiopia and India include KMC training. We will update them to include strategies to initiate and continue KMC at health facilities and plans for follow-up care at home. These study manuals will be used to train care providers in the selected study health facilities. To support health workers in implementing KMC, we will develop job-aids, checklists and data collection forms.

Launch of the KMC implementation program: A one-day workshop will be conducted involving officials and experts from study sites including regional and district health offices, health facilities, and MNCH implementing partners. The workshop will orient stakeholders and strengthen local partnerships for successful implementation of project activities.

Implementation team training: We will provide an intensive training on KMC for health care workers from study facilities and CHWs from the community. Training will involve lectures and hands-on training, including technical updates, counselling skills, follow up care at health facility and at home, and documentation of the KMC services. Trainers will be experienced local paediatricians and faculty from the site-specific institutions and the pool of trainers already available in the state. Training components will include advantages of KMC, appropriate KMC techniques, possible barriers and ways to address them, how to support mothers to initiate and sustain KMC and counselling mothers/family members at discharge. Practical training will be coordinated centrally, at a dedicated KMC unit, initially through simulators and subsequently with mothers and their babies. Refresher training sessions will be held. Follow up with supportive supervision will be set up to ensure that the physicians and nurses translate their learning into implementation at their facilities. The supportive supervision will be initially ensured by the study team.

For peer review only

For peer review only - http://bmjopen.bmj.com/site/about/guidelines.xhtml

The CHWs and their supervisors will be trained on essential newborn care. Master trainers will be involved. The monthly meetings of the CHWs will provide opportunities for follow up re-training and query-solving of problems encountered during their work in the community.

Immediately after training, Objective Structured Clinical Examinations (OSCEs) will be conducted to test trainees' knowledge and skills. Mentors' training will be done at each level of the health system.

Process-monitoring

The program learning team will transition to documenting implementation processes by measuring process indicators. Process indicators for KMC implementation were described in Table 3.

For peer review only

For peer review only - http://bmjopen.bmj.com/site/about/guidelines.xhtml

Table 3. Process Indicators for KMC Implementation

Process	Indicator
Identification of pregnant women	• Proportion of pregnant women identified by CHW in the antenatal period
Exposure to KMC priming interactions in the antenatal period	• Proportion of identified pregnant women who received KMC information in the antenatal period from CHWs (either at home visits or during planned community interactions<br>• Proportion of identified pregnant women who received KMC information during ANC visits at health facilities
Delivery in KMC facility	• Proportion of women who deliver in a KMC facility versus in another facility or at home
Weighed at birth	• Proportion of live born babies who were weighed at birth, overall and by place of birth
Identification of newborns <2000 g	• Proportion of babies identified as <2000 g, overall and by place of birth
Referral	• Proportion of newborns <2000 g who were born at home or in non-KMC facility who were successfully referred to KMC facility for care
Initiation of KMC	• Proportion of newborns <2000 g born in KMC facility who were initiated on KMC<br>• Proportion of newborns <2000 g referred to KMC facility who were initiated on KMC
KMC monitoring	• Proportion of KMC initiated newborns monitored by facility staff according to protocol<br>• Proportion of facility KMC newborns discharged according to criteria; left against medical advice; referred out; died before discharge<br>• Duration of stay in facility
KMC follow-up	• Proportion of newborns discharged from facility receiving KMC who received follow-up per protocol

For peer review only - http://bmjopen.bmj.com/site/about/guidelines.xhtml

Outcome Measurement

The outcome measurement team will evaluate the effectiveness of the model in achieving 80% or higher coverage with KMC among the entire study population as described above in the outcome measurement section.

Evaluation

Effective KMC coverage is defined as the number of newborns receiving KMC divided by the total number of newborns eligible for KMC (<2000 g and stable) in the facilities and in the study population during the evaluation period.

To measure the denominator for effective KMC coverage it is preferred that all facility births be identified by the outcome measurement team during facility visits, review of birth registers, and/or calls to the facilities. In addition, the outcome measurement team will liaise with CHWs to obtain the number of home births and their birth weights.

To calculate the numerator for effective KMC coverage, the outcome measurement team will quantify the number of newborns under 2000 g who received KMC in the study population. All identified newborns under 2000 g will be followed by the outcome measurement team in the facility or at home to collect data at four points i.e. day of initiation of KMC, day of discharge from the KMC facility, seven days post-discharge, and at age 28 days. At each time point, the team will measure KMC based on records, and/or maternal report, if post-discharge.

Timeline

The study time-line is displayed in Figure 4.

INSERT FIGURE 4 HERE

Data Management

Data collected will be linked to participant identification numbers. Each research team will be responsible for data management for its site. The cleaned data set will be sent to WHO at monthly intervals for review and feedback. Data quality assurance methods would include online real-time checks for missing values and inconsistency; random spot checks and data validation will be coupled with independent quarterly audits.

For peer review only - http://bmjopen.bmj.com/site/about/guidelines.xhtml

Oversight

The Maternal, Newborn, Child and Adolescent Health (MCA) Department of WHO, Geneva will create a Steering Committee and be responsible for organizing its meetings. The Steering Committee will be the technical body responsible for the oversight.

DISCUSSION

Despite its scientifically documented benefits, scaling up of KMC has proven an elusive goal for about 40 years. With increased awareness of the magnitude of newborn mortality and the clear interest of the governments of Ethiopia and India in making KMC available to all newborns of birth weight below 2000 g, it is timely to implement this project. It provides a great opportunity to weave KMC within the existing health system and, in collaboration with the government, to find effective models of implementation applicable at scale and sustainable. The findings from this implementation research project will provide inputs to policy-makers to formulate KMC implementation guidance for state or nationwide scale up, if one doesn't yet exist, or to improve existing ones. The findings will have implications for additional actions to strengthen provision of essential newborn care.

Literature on barriers and facilitators of KMC within health facilities is available.[29-31] This project aims to develop models and test them in cycles with an inbuilt iterative learning component and strong government-research partnerships, that will ultimately lead to identification of effective models of scale up in large country-wide settings. While we focus on KMC coverage in our outcomes, our aim is to develop a model that incorporates KMC as an integral component of newborn care so that the KMC scale up is not projected as a vertical program. The experiences from this research will not only inform us on the challenges to scale up KMC and ways to effectively address them but will have the potential to help the design and implementation strategy of future infant and child care programs in a way that their accelerated uptake by health system and the communities is strengthened. The findings of this implementation research will also be useful in other low- and middle-income countries, besides India and Ethiopia.

For peer review only - http://bmjopen.bmj.com/site/about/guidelines.xhtml

Figure legends

Figure 1 – Core framework for KMC implementation

Figure 2 – Logic model for KMC scale-up

Figure 3 – Optimization of implementation model

Figure 4 – Research timeline

Study status

WHO approved protocol: Version 4 - 12 May 2016– Protocol ID: ERC 2716

Study implementation beginning: April 2017

Study end: expected March 2019

Disclaimer

The authors alone are responsible for the views expressed in this article and they do not necessarily represent the views, decisions or policies of the institutions with which they are affiliated.

Contributor's statement page

All authors contributed to the preparation of the manuscript. The specific contributions are listed below:

Conceptualization of the study and first drafting of the manuscript: Abebe Gebremariam Gobezayehu, Mulusew Lijalem, Lynn M Sibley, Grace J Chan, Abiy Seifu Estifano, Damen Haile Mariam, Henok Tadele, Birkneh Tilahun Tadesse, Khalid Aziz, Araya Abrha Medhanyie, Tedros Hailu Abay, Sarmila Mazumder, Ravi Prakash Upadhyay, Nita Bhandari, Prem K. Mony, Suman Rao, Krishnamurthy Jayanna, Arin Kar, Aarti Kumar, Gary Lee Darmstadt, Vishwajeet Kumar, Rajiv Bahl, Cynthia Pileggi-Castro, Jose Carlos Martines

Subsequent revisions of manuscript drafts, completion of information on study settings and methods: John N Cranmer, Abebe Gebremariam Gobezayehu, Mulusew Lijalem Belew, Bizuayehu Gashaw Andualem, Hajira Mohammed Amin, Lyn Sibley, Grace J Chan, Abiy Seifu Estifanos, Addisalem Fikre, Hibret Alemu, Abraham Tariku, Dereje Duguma, Damen Haile Mariam, Henok Tadele, Birkneh Tilahun Tadesse, Anteneh Asefa, Habtamu Beyene, Bogale Worku, Mekonnen Muleta, Thomas Brune, Nalini Singhal, Khalid Aziz, Araya Abrha

For peer review only - http://bmjopen.bmj.com/site/about/guidelines.xhtml

Medhanyie, Tedros Hailu Abay, Selemawit Asfaw Beyene, Fisseha Ashebir Gebregizabher, Marta Yemane Hadush, Ephrem Tekle Lemango, Sarmila Mazumder, Ravi Prakash Upadhyay, Tarun Kumar Madhur, Arun Jadaun, Nita Bhandari, Prem K. Mony, Suman Rao, Krishnamurthy Jayanna, Arin Kar, Mohan HL, Rajini M, Aarti Kumar, Raghav Krishna, Kajal, Gary Lee Darmstadt, Vishwajeet Kumar, Jose Carlos Martines, Rajiv Bahl.

All authors read and approved the final manuscript.

Acknowledgements

We acknowledge the support of Anayda Portela, Daphne McRae and Bertrand Leduc in the preparation of the final manuscript.

REFERENCES

1. Blencowe H, Cousens S, Oestergaard MZ, Chou D, Moller AB, Narwal R, et al. National, regional, and worldwide estimates of preterm birth rates in the year 2010 with time trends since 1990 for selected countries: a systematic analysis and implications. Lancet. 2012; 379:2162–72.
2. Blencowe H, Cousens S, Chou D, Oestergaard M, Say L, Moller AB, et al. Born too soon: the global epidemiology of 15 million preterm births. Reprod Health. 2013;10 Suppl 1:S2.
3. Lawn JE, Davidge R, Paul VK, von Xylander S, de Graft Johnson J, et al. Born too soon: care for the preterm baby. Reprod Health. 2013;10 Suppl 1:S5
4. Kangaroo Mother Care: a practical guide. Department of Reproductive Health and Research, World Health Organization (2003). Available at: http://apps.who.int/iris/bitstream/10665/42587/1/9241590351.pdf
5. Conde-Agudelo A, Díaz-Rossello JL. Kangaroo mother care to reduce morbidity and mortality in low birth weight infants. Cochrane Database of Systematic Reviews 2016, Issue 8. Art. No.: CD002771.DOI: 10.1002/14651858.CD002771.pub4
6. Every newborn: an action plan to end preventable deaths. World Health Organization (2014). Available at: http://apps.who.int/iris/bitstream/10665/127938/1/9789241507448_eng.pdf?ua=1
7. Vesel L, Bergh AM, Kerber KJ, Valsangkar B, Mazia G, Moxon SG, et al. Kangaroo mother care: a multi-country analysis of health system bottlenecks and potential solutions. BMC Pregnancy Childbirth. 2015;15 Suppl 2:S5.
8. Bergh AM, Charpak N, Ezeonodo A, Udani R, van Rooyen E. Education and training in the implementation of kangaroo mother care. S Afr J Child Health. 2012;6(2):38–45.
9. The state of the world's children 2016: a fair chance for every child. United Nations Children's Fund (UNICEF), 2016. Available at: https://www.unicef.org/publications/files/UNICEF_SOWC_2016.pdf
10. Central Statistical Agency (CSA) [Ethiopia] and ICF. 2016. Ethiopia Demographic and Health Survey 2016: Key Indicators Report. Addis Ababa, Ethiopia,and Rockville, Maryland, USA. CSA and ICF. Available at:

For peer review only - http://bmjopen.bmj.com/site/about/guidelines.xhtml

https://www.usaid.gov/sites/default/files/documents/1860/Ethiopia%20DHS%202016%20KIR%20-%20Final%2010-17-2016.pdf
11. The Last Ten Kilometers Project (L10K). Trends in reproductive, maternal, newborn and child health care practices in 115 L10K woredas: Analyses of three rounds of survey data. JSI Research & Training Institute, Inc./The Last Ten Kilometers (L10K) Project; 2015. Available at: http://l10k.jsi.com/Resources/Docs/L10KsRoundIIISurveyFinalReport.pdf
12. Federal Democratic Republic of Ethiopia Ministry of Health. HSTP Health Sector Transformation Plan 2015/16 – 2019/20 (2008–2012 EFY); 2015. Available at: https://www.globalfinancingfacility.org/sites/gff_new/files/Ethiopia-health-system-transformation-plan.pdf
13. Jackson R, Hailemariam A. The role of Health Extension Workers in linking pregnant women with health facilities for delivery in rural and pastoralist areas of Ethiopia. Ethiop J Health Sci. 2016;26(5):471-478.
14. Ethiopia- Population and Housing Census 2007; IPUMS subset. Available at: http://microdata.worldbank.org/index.php/catalog/2747
15. LSMS- Integrated surveys on agriculture; Ethiopia socioeconomic survey (ESS) 2015/2016. Central Statistical Agency and Living Standards Measurement Study (LSMS), World Bank.
16. Sankar MJ, Neogi SB, Sharma J, Chauhan M, Srivastava R, Prabhakar PK, et al. State of newborn health in India. J Perinatol. 2016; 36(Suppl 3): S3–S8.
17. Bhilwar M, Upadhyay RP, Yadav K, Kumar R, Chinnakali P, Sinha S et al. Estimating the burden of 'weighing less': A systematic review and meta-analysis of low birth-weight in India. Natl Med J India. 2016;29(2):73-81.
18. India Newborn Action Plan (INAP). Child Health Division, Ministry of Health and Family Welfare, Government of India. September 2014. Available at: https://www.newbornwhocc.org/INAP_Final.pdf
19. Kangaroo mother care and optimal feeding of low birth weight: Operational guidelines. Child Health Division. Ministry of Health and Family Welfare, Government of India. September 2014. Available at: http://nhm.gov.in/images/pdf/programmes/childhealth/guidelines/Operational_Guidelines-KMC_&_Optimal_feeding_of_Low_Birth_Weight_Infants.pdf
20. Census (2011). Office of the Registrar General and Census Commissioner. Ministry of Home Affairs, Government of India. Available at: http://www.censusindia.gov.in/2011census/PCA/PCA_Highlights/pca_highlights_file/India/Chapter-1.pdf
21. Census of India 2011. District Census Handbook, Sonipat. Directorate of Census Operations, Haryana. Available at: http://www.censusindia.gov.in/2011census/dchb/0608_PART_B_DCHB_SONIPAT.pdf
22. International Institute for Population Sciences (IIPS), 2014. District Level Household and Facility Survey (DLHS-4), 2012-13: India. Haryana; District fact sheet, Sonepat: Mumbai: IIPS. Available at: https://nrhm-mis.nic.in/DLHS4/State%20and%20District%20Factsheets/.../Sonipat.pdf
23. State Fact Sheet-Haryana; National Family Health Survey 2015-2016. Available at: http://rchiips.org/NFHS/pdf/NFHS4/HR_FactSheet.pdf
24. Sample registration system (SRS) Bulletin; Registrar General of India (2016). Available at: http://www.censusindia.gov.in/vital_statistics/SRS_Bulletin_2015.pdf
25. PHFI, AIIMS, and SC- State of India's Newborns (SOIN) 2014- a report. (Eds)

For peer review only - http://bmjopen.bmj.com/site/about/guidelines.xhtml

Zodpey S and Paul VK. Public Health Foundation of India, All India Institute of Medical Sciences and Save the Children. New Delhi, India. Available at: https://www.newbornwhocc.org/SOIN_PRINTED%2014-9-2014.pdf

26. Mony PK, Jayanna K, Bhat S, Rao SV, Crockett M, Avery L, et al. Availability of emergency neonatal care in eight districts of Karnataka state, southern India: a cross-sectional study. *BMC Health Serv Res* 2015; 15(1): 461
27. Annual Health Survey fact sheet; Uttar Pradesh (2012-13). Vital Statistics Division. Office of the Registrar General and Census Commissioner, Ministry of Home Affairs, Government of India. Available at: http://www.censusindia.gov.in/vital_statistics/AHSBulletins/AHS_Factsheets_2012-13/FACTSHEET-UTTAR_PRADESH.pdf
28. Michie, S., M.M. van Stralen, and R. West. The behaviour change wheel: a new method for characterising and designing behaviour change interventions. Implement Sci. 2011. 6: 42
29. Seidman G, Unnikrishnan S, Kenny E, Myslinski S, Cairns-Smith S, Mulligan B, et al. Barriers and enablers of Kangaroo Mother Care practice: a systematic review. PLOS ONE. 2015, 10(5):e0125643.
30. Chan GJ, Labar AS, Wall S, Atun R. Kangaroo mother care: a systematic review of barriers and enablers. Bulletin of the World Health Organization. 2016, 94(2):130-141J.
31. Smith ER, Bergelson I, Constantian S, Valsangkar B, Chan GJ. Barriers and enablers of health system adoption of kangaroo mother care: a systematic review of caregiver perspectives. BMC Pediatr. 2017; 17: 35.

For peer review only

For peer review only - http://bmjopen.bmj.com/site/about/guidelines.xhtml

	**Pre-KMC Facility**	**KMC Facility**	**Post-KMC Facility**
Health workers	Community health workers	Health facility staff: midwives, nurses, medical officer in charge, obstetricians, paediatricians	Community health workers
Activities	- Pregnancy surveillance<br>- Antenatal care visits<br>- Encouragement of facility births<br>- Birth notification<br>- Weigh all newborns<br>- Support cultural norms endorsing KMC<br>- Referral of newborns <2000 g to a KMC facility	- Weigh all newborns<br>- Accept referrals<br>- **Initiate KMC** for all newborns <2000 g<br>- Maintain KMC<br>- Breastfeeding support<br>- Quality Improvement to ensure effective KMC	- Scheduled home visits within 24 hours of discharge and on days of life 7 and 28<br>- **KMC continuation** at home<br>- Breastfeeding support<br>- Identification and referral of sick newborns or newborns unable to continue KMC
Outputs	- Identify KMC eligible newborns (<2000 g)<br>- Get all newborns <2000 g to KMC facility	- Effective KMC initiated and maintained for all newborns <2000 g	- Effective KMC continued at home

Health system strengthening

- Linkage of KMC facilities and communities
- Referral System to identify KMC eligible (<2000 g) and/or sick newborns, and facilitate their transfer from the community to KMC facility

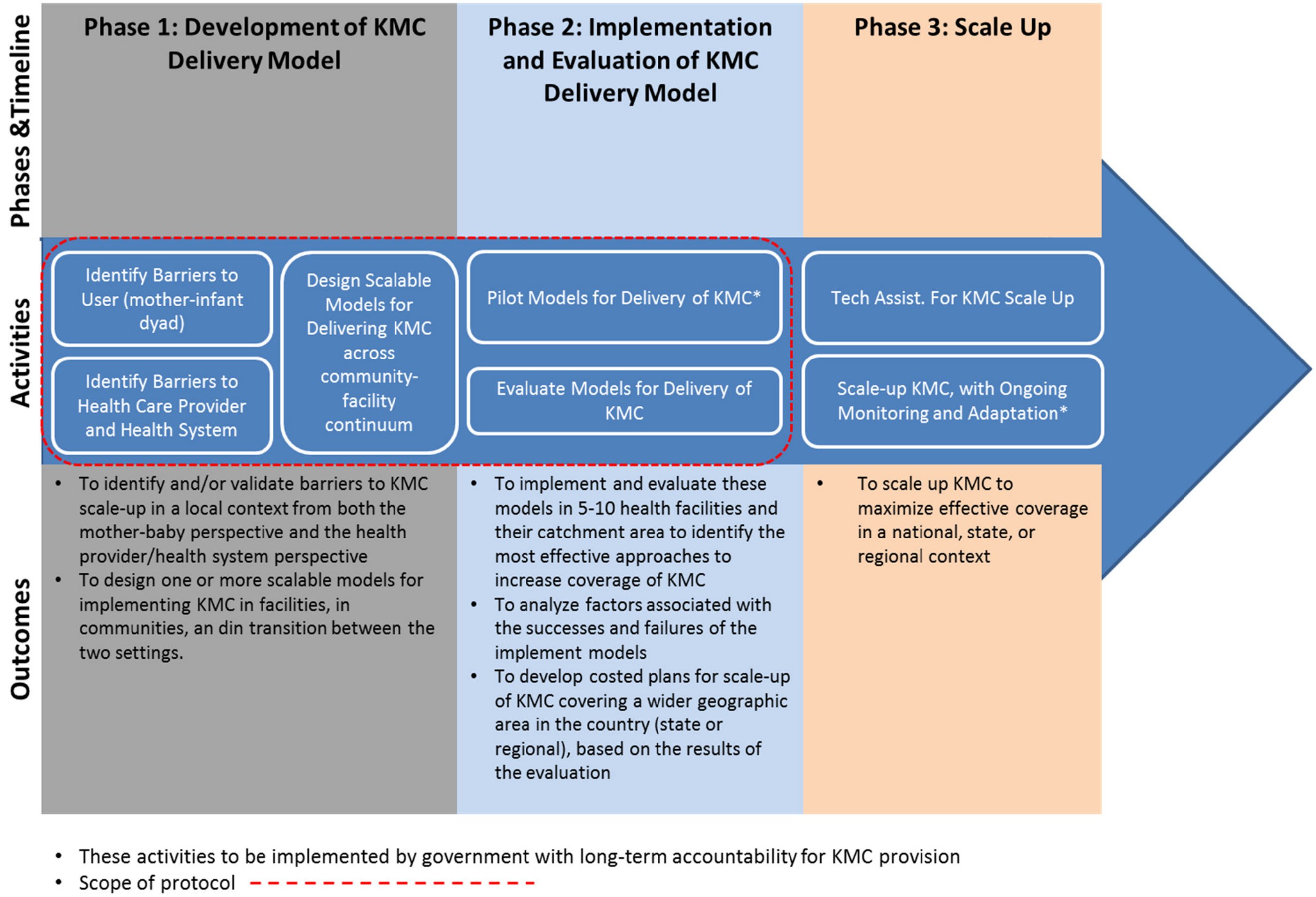
Phases &Timeline
Activities
Outcomes
Phase 1: Development of KMC Delivery Model
Phase 2: Implementation and Evaluation of KMC Delivery Model
Phase 3: Scale Up
Identify Barriers to User (mother-infant dyad)
Identify Barriers to Health Care Provider and Health System
Design Scalable Models for Delivering KMC across community-facility continuum
Pilot Models for Delivery of KMC*
Evaluate Models for Delivery of KMC
Tech Assist. For KMC Scale Up
Scale-up KMC, with Ongoing Monitoring and Adaptation*
• To identify and/or validate barriers to KMC scale-up in a local context from both the mother-baby perspective and the health provider/health system perspective
• To design one or more scalable models for implementing KMC in facilities, in communities, an din transition between the two settings.
• To implement and evaluate these models in 5-10 health facilities and their catchment area to identify the most effective approaches to increase coverage of KMC
• To analyze factors associated with the successes and failures of the implement models
• To develop costed plans for scale-up of KMC covering a wider geographic area in the country (state or regional), based on the results of the evaluation
• To scale up KMC to maximize effective coverage in a national, state, or regional context
• These activities to be implemented by government with long-term accountability for KMC provision
• Scope of protocol

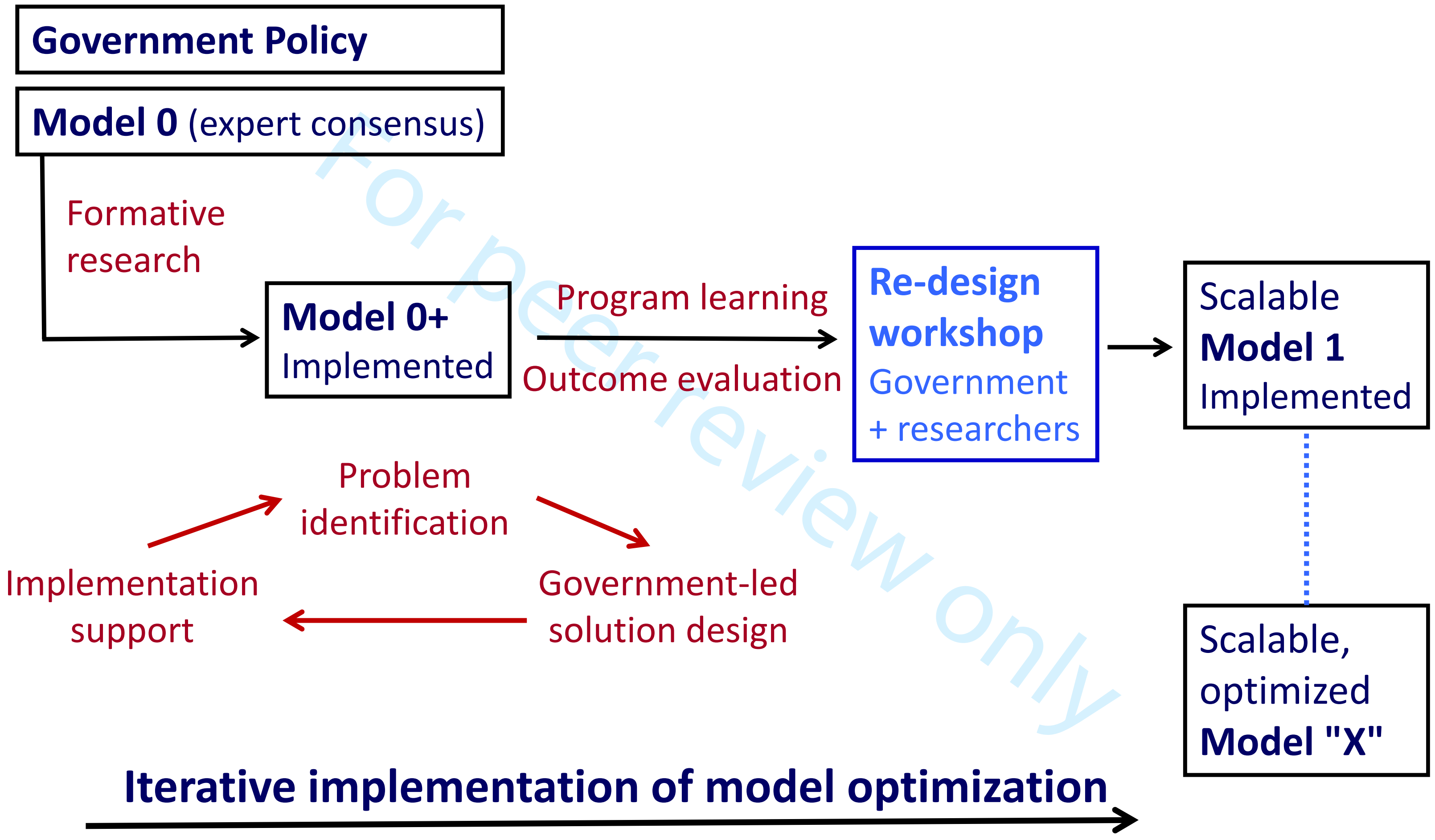

For peer review only - http://bmjopen.bmj.com/site/about/guidelines.xhtml

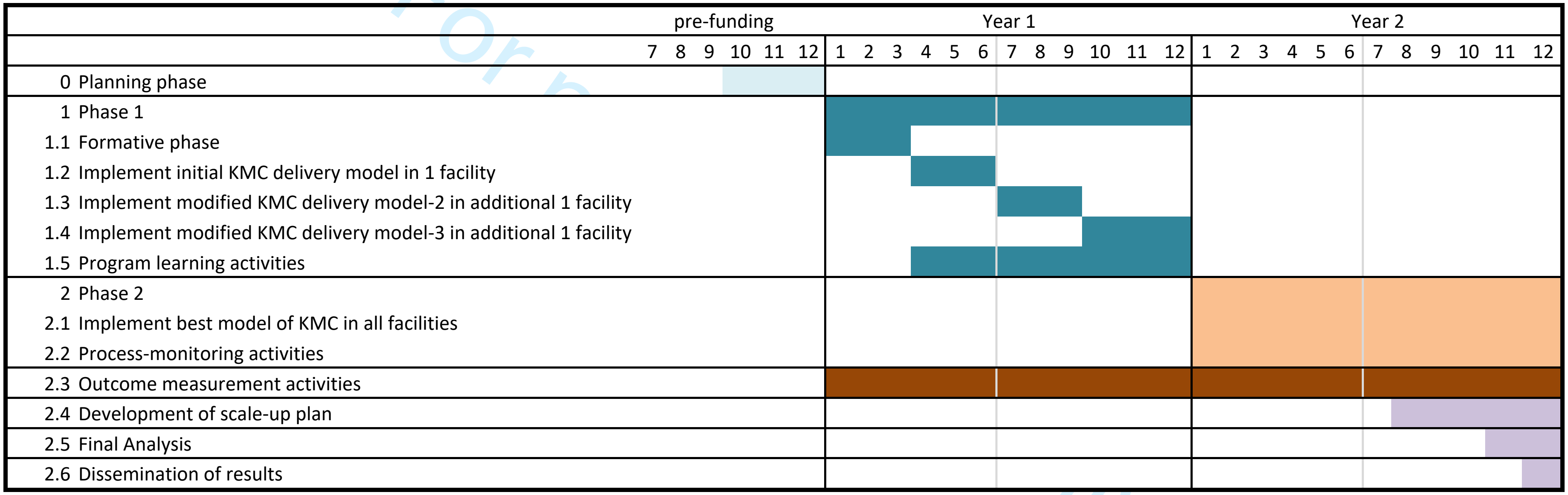

For peer review only - http://bmjopen.bmj.com/site/about/guidelines.xhtml
